# Melanopsin Contributions to Irradiance Coding in the Thalamo-Cortical Visual System

**DOI:** 10.1371/journal.pbio.1000558

**Published:** 2010-12-07

**Authors:** Timothy M. Brown, Carlos Gias, Megumi Hatori, Sheena R. Keding, Ma'ayan Semo, Peter J. Coffey, John Gigg, Hugh D. Piggins, Satchidananda Panda, Robert J. Lucas

**Affiliations:** 1 Faculty of Life Sciences, University of Manchester, Manchester, United Kingdom; 2 Institute of Ophthalmology, University College London, London, United Kingdom; 3 The Salk Institute for Biological Studies, La Jolla, California, United States of America; University of Washington, United States of America

## Abstract

Photoreception in the mammalian retina is not restricted to rods and cones but extends to a subset of retinal ganglion cells expressing the photopigment melanopsin (mRGCs). These mRGCs are known to drive such reflex light responses as circadian photoentrainment and pupillomotor movements. By contrast, until now there has been no direct assessment of their contribution to conventional visual pathways. Here, we address this deficit. Using new reporter lines, we show that mRGC projections are much more extensive than previously thought and extend across the dorsal lateral geniculate nucleus (dLGN), origin of thalamo-cortical projection neurons. We continue to show that this input supports extensive physiological light responses in the dLGN and visual cortex in mice lacking rods+cones (a model of advanced retinal degeneration). Moreover, using chromatic stimuli to isolate melanopsin-derived responses in mice with an intact visual system, we reveal strong melanopsin input to the ∼40% of neurons in the LGN that show sustained activation to a light step. We demonstrate that this melanopsin input supports irradiance-dependent increases in the firing rate of these neurons. The implication that melanopsin is required to accurately encode stimulus irradiance is confirmed using melanopsin knockout mice. Our data establish melanopsin-based photoreception as a significant source of sensory input to the thalamo-cortical visual system, providing unique irradiance information and allowing visual responses to be retained even in the absence of rods+cones. These findings identify mRGCs as a potential origin for aspects of visual perception and indicate that they may support vision in people suffering retinal degeneration.

## Abstract 2

Neurophysiological and anatomical studies identify melanopsin expressing retinal ganglion cells (mRGCs) as a major source of information in the mouse visual system.

## Author Summary

The ability to convert light into electrical signals in the mammalian retina has traditionally been thought to occur through only two types of photoreceptors, the rods and cones. However, this ability also extends to a small number of neurons expressing the protein melanopsin. To date these melanopsin retinal ganglion cells (mRGCs) have been thought to act as photoreceptors primarily for those brain regions regulating subconscious responses to ambient light, such as pupil size. In this study, we provide evidence that mRGCs also contribute extensively to conventional visual pathways. We use a newly developed strategy to characterize the brain regions to which these cells project and then directly monitor visually evoked neural activity in these target regions. We find that mRGCs allow the mouse visual system to faithfully encode ambient illumination over at least a million-fold range and to continue to show light responses even in the absence of all rods and cones. These data reveal a new role for mRGCs during normal vision and suggest that mRGCs could make a significant contribution to assessing brightness and supporting vision even in people with advanced retinal degeneration.

## Introduction

Until 10 years ago it was considered self-evident that all aspects of mammalian vision originate with light detection by either rods or cones. This view has changed with the discovery of a 3^rd^ photoreceptor class [1]—the so-called melanopsin retinal ganglion cells (mRGCs). These mRGCs are able to respond to light even in complete isolation thanks to the presence of melanopsin, an opsin-based photopigment.

Until now, the discovery of mRGCs has had the most impact on our understanding of such sub-cortical light responses as circadian entrainment and the pupil light reflex. We now know that mRGCs integrate input from the outer retina with their own intrinsic light response to encode ambient light intensity (irradiance or illuminance) for these and other reflex responses [2]–[11]. By contrast, there has been little consideration of melanopsin's potential to contribute to the physiology or function of conventional visual pathways and little effort to incorporate these newly discovered photoreceptors into models of visual perception.

This omission has reflected the balance of published evidence that mRGC axons are largely segregated from the thalamo-cortical visual pathway. Thus, while Dacey et al. reported back-labeling mRGCs from the primate visual thalamus [12], more extensive analysis of the mRGC projection pattern in rodents [13]–[15] suggested that their input to this part of the brain was almost exclusively restricted to the ventral LGN and intergeniculate leaflet, rather than the dLGN which is the site of thalamo-cortical projection neurons.

It has since become clear, however, that the *Opn4^tauLacZ^* transgenic reporter line employed in the most comprehensive of those rodent studies has incomplete penetrance and labels only around 50% of melanopsin-expressing retinal ganglion cells [11],[13],[16]–[17]. The successfully labeled neurons represent a distinct anatomical subtype (called M1) whose dendrites are restricted to the outer sub-lamina of the inner plexiform layer. The projection pattern of the remaining mRGC classes (which have dendrites in the inner sublamina) has only been very recently addressed [18]. There is thus renewed uncertainty regarding the degree to which mRGCs influence the dLGN and whether they could make a direct contribution to aspects of conventional vision.

Here, we employ a Cre-recombinase based reporter line to trace the projection pattern of the full mRGC population. This reveals a substantial innervation of the dLGN. We continue to assess the physiological consequences of this anatomical organization using multichannel extracellular electrophysiology and brain imaging in the visual thalamus and cortex. These experiments reveal an essential role for mRGCs in encoding irradiance for the central pathways of conventional vision.

## Results

### mRGCs Innervate All Major Retinorecipient Regions

**Figure 1:**
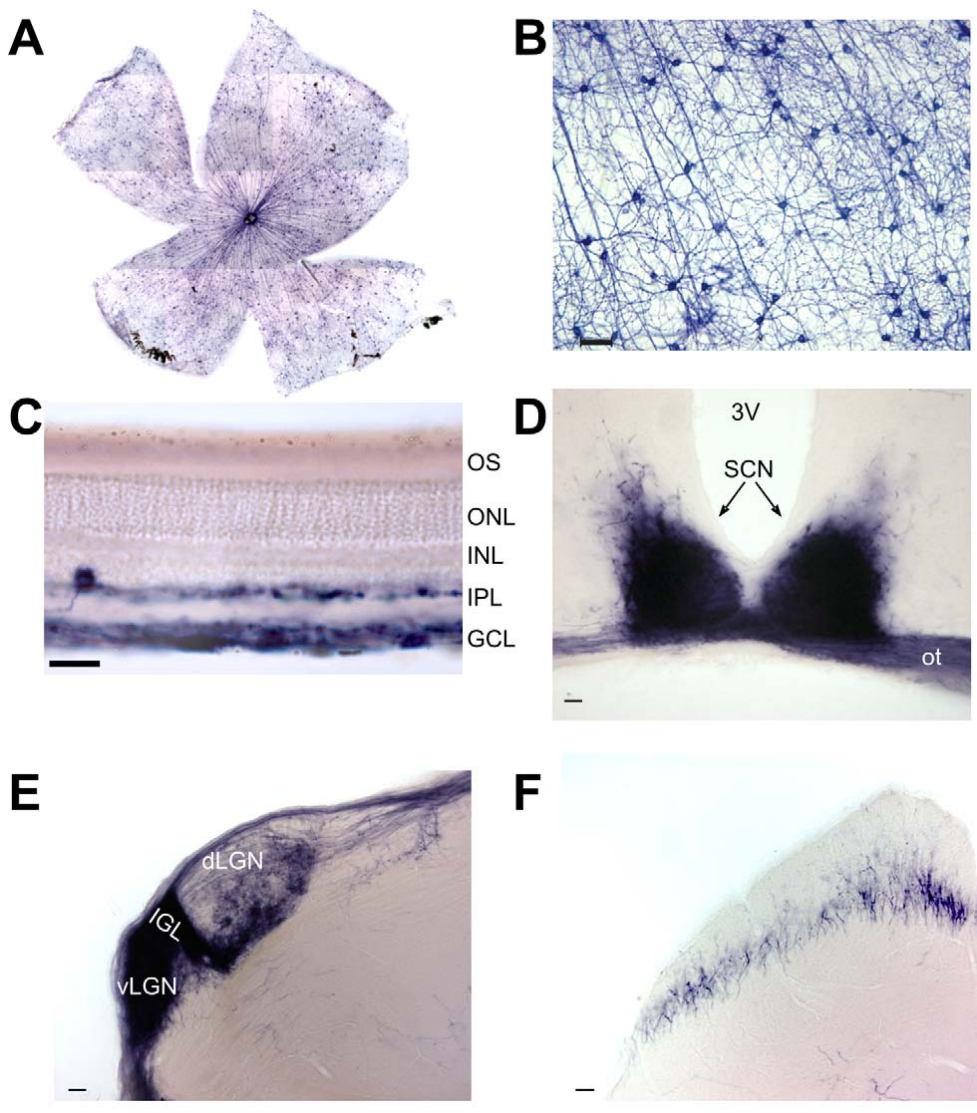
Genetic labeling of melanopsin RGCs and their projections. (A) Alkaline phosphatase (AP) stained mRGCs in *Opn4^Cre^;Z/AP* mice are uniformly distributed across the retina (1,556±72; mean ± SD, *n* = 4). (B) AP labels the soma, dendrites, and axons of mRGCs. (C) Labeled cell bodies are restricted to the ganglion cell layer (GCL) and inner nuclear layer (INL), with dendrites in both sublaminae of the inner plexiform layer (IPL). (D–F) AP-stained coronal brain sections demonstrate dense mRGC innervation of the suprachiasmatic nuclei (SCN, D), intergeniculate leaflet (IGL, E), ventral and dorsal lateral geniculate nuclei (v/dLGN, E), and sparse innervation of the superior colliculus (F). 3V, third ventricle; ot, optic tract. Scale bars represent 50 µm (B, D–F) and 25 µm (C).

To comprehensively label the mRGCs and their central projections we crossed mice in which a Cre-recombinase transgene was knocked into the melanopsin locus (*Opn4^Cre/Cre^* [11]) with suitable reporter lines: *Z/AP* or *Z/EG* [19],[20]. In this strategy, appearance of the reporter protein (human alkaline phosphatase (AP) or green fluorescent protein) is dependent on the expression of Cre from the native melanopsin promoter. Importantly, however, reporter gene expression is independent of heterogeneity in activity of the native melanopsin promoter because it is driven by a ubiquitous β-actin promoter. This approach therefore has the potential to achieve a more complete coverage of the mRGC population than possible with the established *Opn4^tauLacZ^* reporter line [13]. We found that, indeed, >1,500 retinal ganglion cells per retina were labeled in these Cre-recombinase-based lines (Figure 1A,B). This is equivalent to the number of cells immunopositive for melanopsin but around twice the number detected in *Opn4^tauLacZ^* mice [13],[16]. Indeed, the appearance of reporter protein in both outer and inner sublaminae of the inner plexiform layer indicated that not only M1 but also other mRGC classes had been labeled (Figure 1C). Accordingly, 90% of GFP expressing cells in our Z/EG reporter line retina were also labeled by an antibody raised against the N-terminus of melanopsin, confirming that these cells were mRGCs (Figure S1). The remaining GFP-positive soma were restricted to the ganglion cell layer and proximal zone of the inner nuclear layer and presumably either reflect cells with very low melanopsin expression or cells that transiently expressed melanopsin during development.

We then used the *Opn4^Cre^;Z/AP* mice to trace the axonal projections of the labeled mRGCs (Figure 1D–F and Figure S2). The observed projection pattern was similar to that reported very recently using a similar strategy [18] and much more extensive than originally described [13]. AP positive axons could be traced along the optic nerve/tract and targeted not only nuclei responsible for reflex light responses (the SCN, IGL, and OPN) but also those implicated in pattern vision. Thus, significant contralateral projections of the mRGCs were observed in both ventral and dorsal aspects of the LGN and (to a lesser extent) in the superior colliculus (SC).

### Widespread Thalamo-Cortical Activation in Mice Lacking Rods and Cones

**Figure 2:**
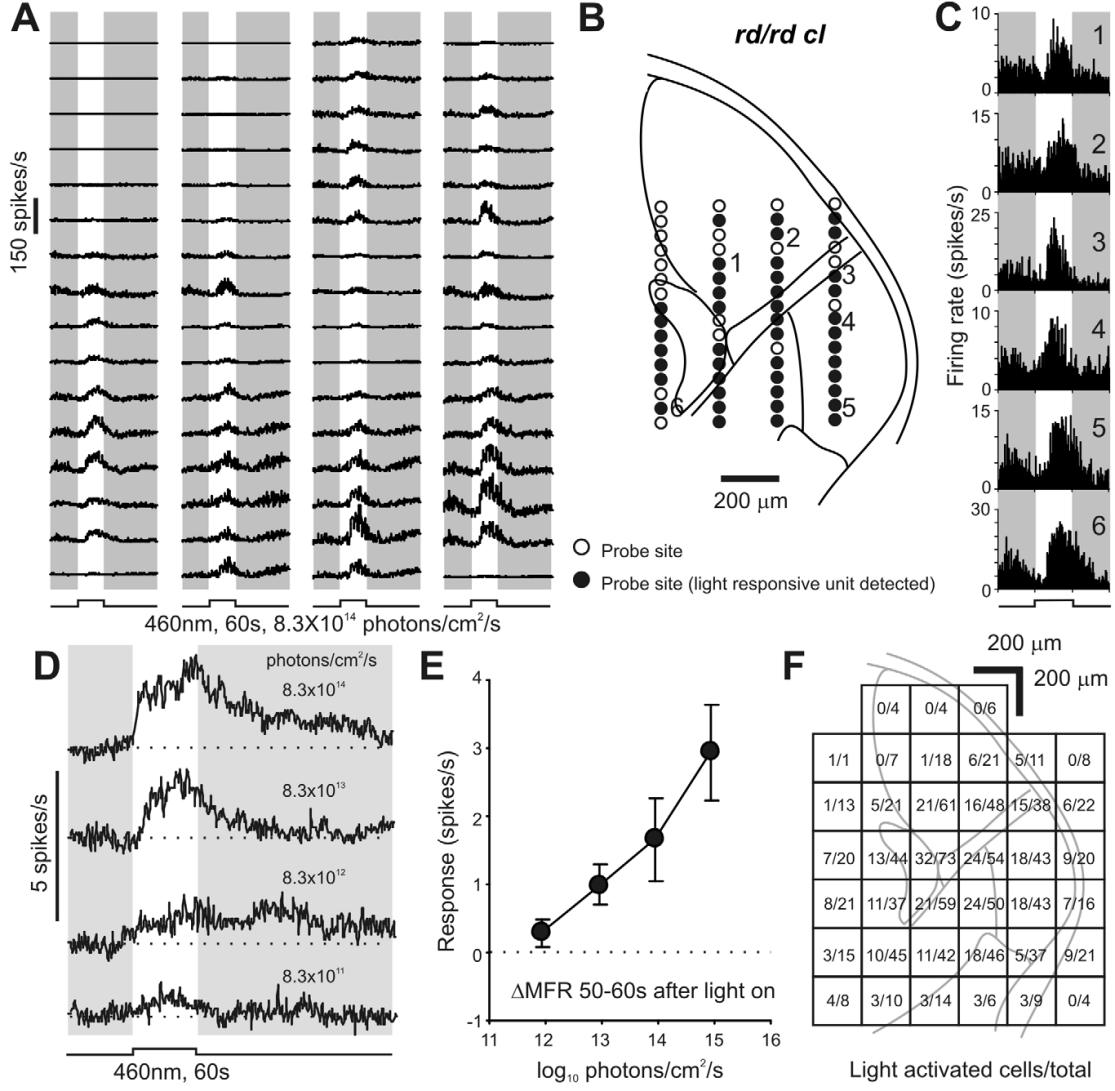
Melanopsin stimulation activates neurons throughout the lateral geniculate. (A) Multiunit responses in the LGN of an *rd/rd cl* mouse showing widespread light activation. Traces are average of four responses and correspond to probe sites shown superimposed on a schematic of the mouse LGN and surrounding thalamus in panel B. Filled circles in (B) indicate probes sites at which a light responsive single unit could be isolated. (C) Representative light responsive single unit firing profiles (numbered sites in B) identified in the dLGN (1,2), IGL/vLGN (3–5), and medial thalamus (6). (D) Average response waveform of light activated cells (*n* = 344 from 18 mice) to 60 s light pulses of increasing intensity showing kinetics characteristic of the melanopsin photoresponse. (E) Change in mean firing rate (ΔMFR) ± SEM for the data in (D) during the last 10 s of the light pulse. (F) Anatomical distribution of light responsive cells relative to the total number detected in each 200 µm^2^ grid across all experiments (*n* = 1,051).

mRGCs survive rod and cone loss to drive reflex light responses in advanced retinal degeneration. To determine whether they could also activate the thalamo-cortical visual projection responsible for visual perception under these circumstances, we set out to record light responses in mice lacking all rods and cones (*rd/rd cl*). These animals show complete rod and cone degeneration [21]–[23] but intact melanopsin signaling and have retinal ganglion cells with normal projection patterns (Figure S3). Using multichannel, multiunit recordings we surveyed light responses to a stimulus designed to activate melanopsin (60 s, 460 nm, 8.3×10^14^ photons/cm^2^/s) in the contralateral visual thalamus of this genotype (Figure 2A,B). We found that widespread light responses across the LGN (including both ventral and dorsal subdivisions) survived rod and cone loss in these mice (Figure 2C,F).

Spike sorting of the multiunit activity resolved 1,051 single units over the 18 *rd/rd cl* mice used for these experiments. A large proportion of these (*n* = 344) were strongly excited by light (Figure 2D), while a small minority were suppressed (*n* = 63, not shown; presumably secondary to activation of inhibitory interneurons). The predominant, light activated, response had relatively low sensitivity (Figure 2D,E; threshold ∼10^12^ photons/cm^2^/s) and extremely sluggish kinetics (earliest response:1.5–2 s; time to peak: 34.2±0.7 s; duration at halfmax firing rate: 53.1±2.3 s), matching the known characteristics of melanopsin phototransduction [12],[24]–[27]. Moreover, the anatomical distribution of these responses (Figure 2F) was consistent with the newly described mRGC input to the contralateral LGN (Figure 1E; [18]), encompassing the ventral LGN/IGL and the medioventral half of the dLGN. Across these regions there were no clear differences in the sensitivity or kinetics of the responses (Figure S4) and within each area the number of responding cells was surprisingly large, with 30%–40% of resolved single units showing photoactivation.

**Figure 3:**
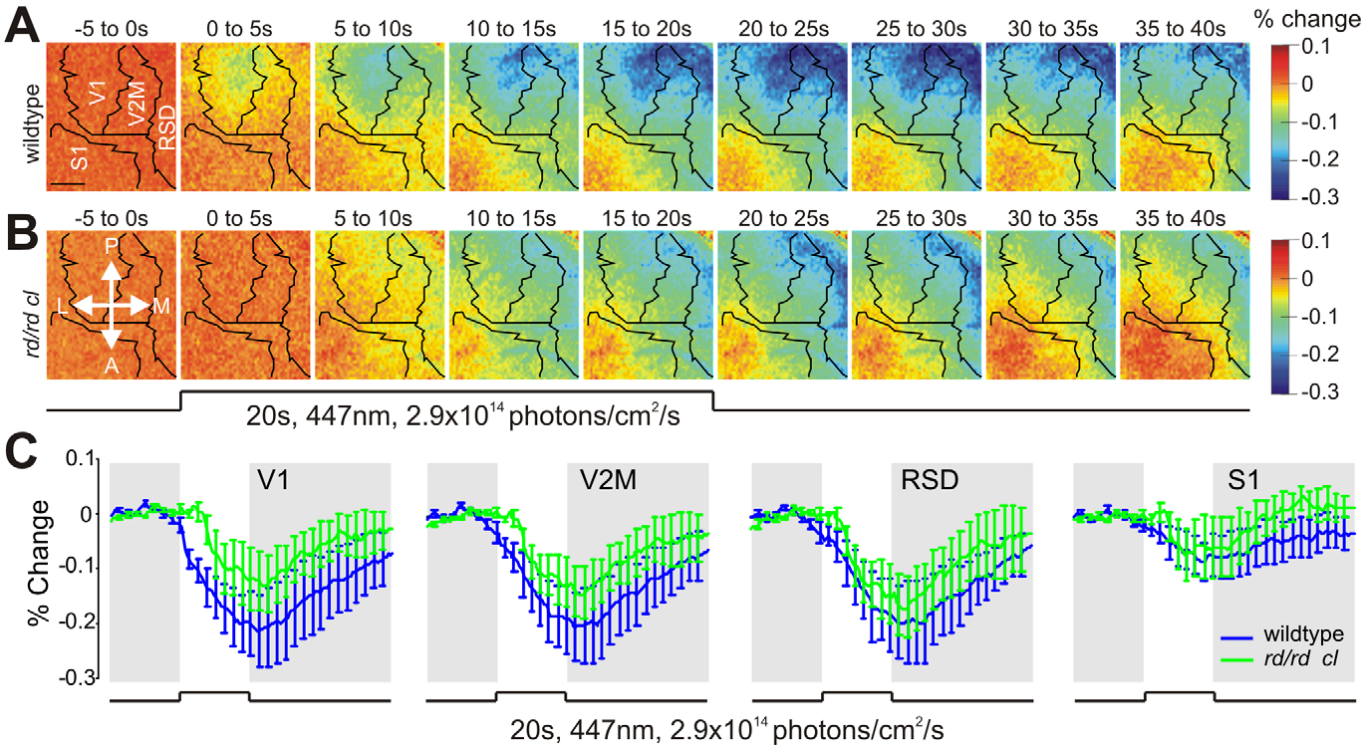
Melanopsin activates the visual cortex. (A,B) Average pseudo-colored maps of cortical activity in response to 20 s light stimulation in the *rd/rd cl* and wildtype mice (both *n* = 4). Green/blue areas on map indicate a decrease in the optical imaging signal, corresponding to neuronal activation. Scale bar represents 1 mm. P, Posterior; M, Medial; A, Anterior; L, Lateral. (C) Time course of the optical imaging signal at different cortical regions (mean ± SEM) in *rd/rd cl* and wildtype mice. At all but the earliest timepoints the light response *rd/rd cl* mice accounted for a large proportion of the wildtype response. RSD, retrosplenial dysgranular cortex; S1, somatosensory area 1; V1, visual area 1; V2M, visual area 2 medial.

The widespread activation of dLGN neurons by mRGCs in *rd/rd cl* mice raises the question of whether these signals propagate to higher visual centers. To test this possibility, we assessed the cortical response of *rd/rd cl* and wildtype mice to blue light stimulation (447 nm, 20 s, 2.9×10^14^ photons/cm^2^/s) using intrinsic optical imaging. This technique exploits changes in reflected light induced by local neural activity to map cortical space [28]–[31], with the amplitude of the signal inversely correlated to the degree of local excitation. In wildtype mice, a cortical response appeared within 5 s of stimulus onset in an ellipsoidal region of the cortex corresponding to V1 (Figure 3A). Subsequently, the response extended to surrounding cortical areas, forming a band spanning the posterior aspect of V2M and retrosplenial dysgranular cortex (RSD; an area which actively processes visual information and is implicated in spatial navigation [32],[33]). The amplitude of these responses increased monotonically, peaking approximately ∼20–25 s after stimulus onset in each region, before returning slowly to baseline (Figure 3A,C).

With the exception of the earliest response (<5 s) in V1, this strong light activation across V2M, RSD, and posterior/medial aspects of V1 was retained in *rd/rd cl* mice (Figure 3B,C). The implication that melanopsin signals reach these higher visual centers is supported by the sluggish kinetics of this cortical response and by a recent report of melanopsin-dependent c-fos induction in the mouse visual cortex [18]. The appearance of the *rd/rd cl* light response within a distinct spatial domain is interesting but hard to interpret given the lack of a consensus regarding the functional organization of the extrastriate mouse visual cortex [30],[34]–[36].

### Melanopsin Signals in the Intact Visual System

The appearance of light responses in the thalamus and cortex of *rd/rd cl* mice confirms that mRGCs can regulate primary visual centers. We next turned our attention to the role of melanopsin in animals with an intact visual system. In order to achieve this goal, we first considered how to isolate the melanopsin component of any visual response. The high threshold sensitivity of melanopsin photoreception (Figure 2; [12],[24]–[27]) provides a clear distinction from rods, which saturate at moderate light intensities [37]–[40]. To separate cone and putative melanopsin activity at higher irradiances, we employed red cone knock-in mice (*Opn1mw^R^*; [41]). These animals have a fully intact visual system and retinal ganglion cells with normal projection patterns (Figure S3) but exhibit a long-wavelength shift in cone spectral sensitivity that allows the cone component of any response to be identified by its enhanced sensitivity to red light [42].

**Figure 4:**
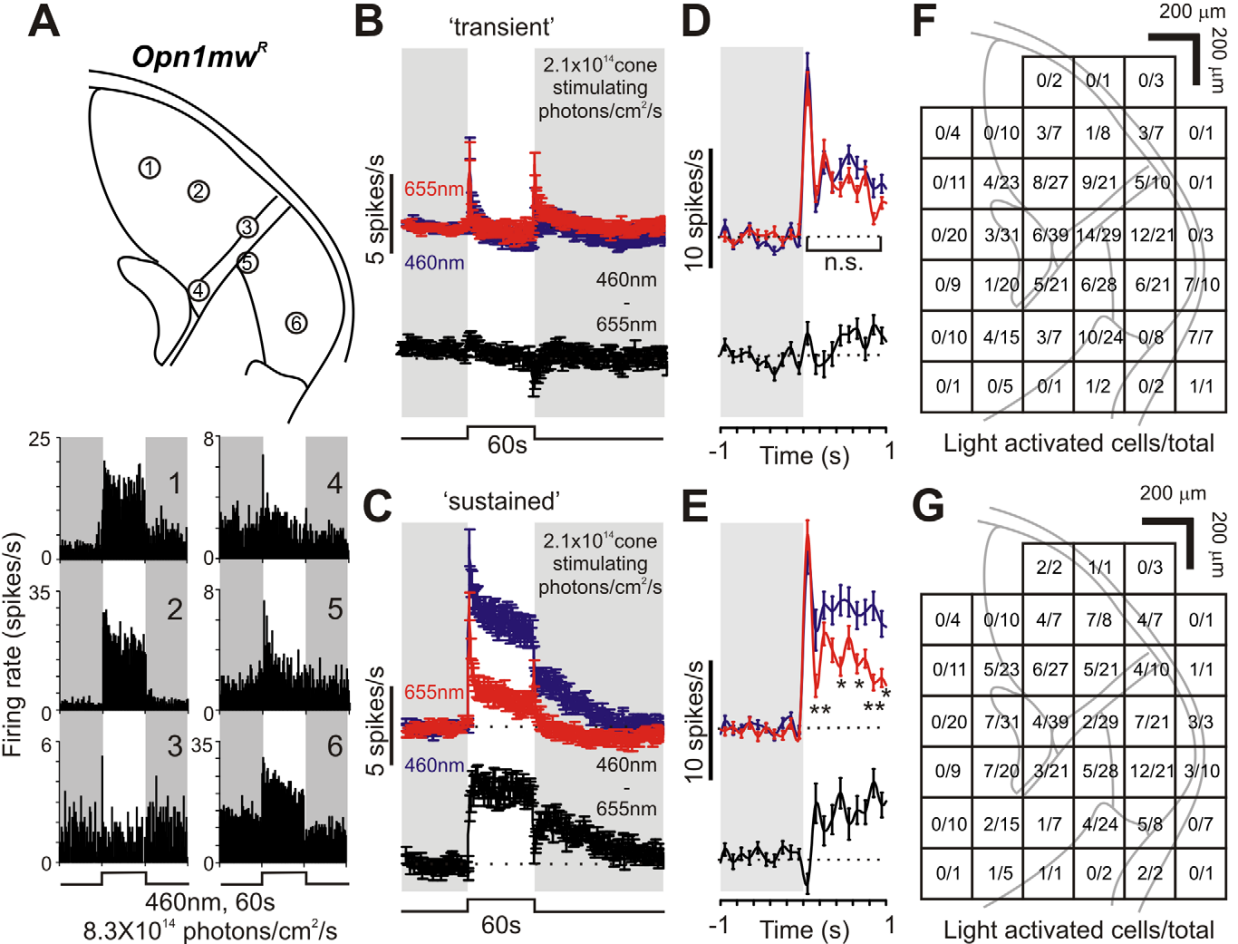
Sustained responses in the lateral geniculate at high irradiances are not driven by cones. (A) Responses of six cells recorded from a representative red cone knockin mouse (*Opn1mw^R^*; multiunit data in Figure S5) to 60 s blue light pulses. A subset maintained high firing rates throughout light exposure (cell 1,2,6; “sustained”), while others showed only “transient” responses (cell 3–5). Upper panel represents the probe sites of cells 1–6. (B,C) Average (± SEM) response of “transient” (B) and “sustained” (C) cells (*n* = 134 and 114, respectively, from 10 mice) to 460 nm/655 nm light pulses isoluminant for cones. Black traces represent the subtraction of the 460 nm and 655 nm responses. Responses to 460 nm and 655 nm light were essentially identical in “transient” cells, whereas “sustained” cells consistently exhibited enhanced responses to 460 nm, consistent with a strong melanopsin input. (D,E) Expansion of the initial “On” transient from (B) and (C) at higher temporal resolution (bin size  = 100 ms), responses of “transient” cells (D) were not significantly different while sustained cells (E) showed enhanced short wavelength responses within 100–200 ms following stimulation (paired *t* tests, n.s. *p*>0.05, * *p*<0.05; ** *p*<0.01). (F,G) Anatomical distribution of “transient” (F) and “sustained” (G) cells relative to the total number of cells detected (based on 542 units). Both cell types were found in approximately equal proportions in both dorsal and ventral portions of the LGN.

Long duration, bright blue stimuli (460 nm, 60 s, 8.3×10^14^ photons/cm^2^/s) elicited robust and sustained increases in multiunit firing across the dorsal and ventral LGN of *Opn1mw^R^* mice (Figure S5). Spike sorting of these data revealed a reproducible light response in 248/542 identifiable single units (from 10 mice). In common with previous work in many mammalian species [43]–[47], we found that these light responsive units fell into distinct “sustained” and “transient” response types (Figure 4). Both showed an initial large increase in firing shortly after light on. Following this, the firing rate rapidly decayed to basal levels in the “transient” group (Figure 4B,D) but remained elevated throughout light stimulation in “sustained” cells (Figure 4C,E). Transient cells typically showed a second brief increase in spike rate following light off. Such “Off” responses were also seen in “sustained” cells although in many cases it was hard to separate them from the persistent response component which typically took 10 s of seconds to return to baseline following light off. Both cell types were found across dorsal and ventral portions of the LGN (Figure 4F,G) in approximately equal numbers (114 and 134 cells, respectively, out of 542 cells recorded from 10 mice).

To isolate melanopsin's contribution to these responses we compared them with those elicited by a long-wavelength stimulus isoluminant for red cones (655 nm, 60 s, 2.6×10^15^ photons/cm^2^/s). At this intensity, both red and blue stimuli are >6 orders of magnitude above the threshold for rod based vision in mice [48] and should be sufficiently bright to elicit a maximal response from rods. Given that the two stimuli have equivalent effects on cones, any difference in response should therefore reflect the contribution of melanopsin (which is essentially insensitive to the longer wavelength). Consistent with these predictions, “transient” cells (whose response phenotype is inconsistent with the characteristics of melanopsin phototransduction) displayed equivalent responses to the two wavelengths (Figure 4B,D; repeated measures two-way ANOVA: Wavelength, F_1,133_ = 1.144, *p*>0.05; Time_0–60 s_, F_59,75_ = 3.829, *p*<0.001; Interaction, F_59,75_ = 1.353, *p*>0.05) confirming that their activity could be explained solely by the influence of outer retinal photoreceptors.

In contrast, all neurons with a “sustained” phenotype were much more responsive to the shorter wavelength (Figure 4C,E; repeated measures two-way ANOVA: Wavelength, F_1,113_ = 87.007, *p*<0.001; Time_0–60 s_, F_59,55_ = 3.705, *p*<0.001; Interaction, F_59,55_ = 3.92, *p*<0.001), suggesting a strong melanopsin influence. 655 nm stimuli did excite these neurons and was even able to maintain elevated discharge rates throughout stimulus presentation, indicating a contribution from cones and/or rods to both transient and, to a lesser extent, sustained phases of the response. Nevertheless, the amplitude of the spiking response to 460 nm was markedly enhanced (Figure 4C), revealing that these “sustained” neurons also receive substantial melanopsin input. As mRGCs receive excitatory input from the outer retina [2]–[5], a portion of the rod/cone contribution to “sustained” excitation in the LGN is presumably routed via this ganglion cell class. However, it seems likely that most (perhaps all) of these thalamic neurons also receive input from more conventional “sustained” ganglion cell classes [49].

The very earliest responses of the “sustained” units were similar at the two wavelengths, consistent with the view that melanopsin phototransduction is too sluggish to influence this aspect of the response (Figure 4E). However, firing was clearly enhanced at the shorter wavelength within 100–200 ms of light on (paired *t* test, *p*<0.01), suggesting that the extremely slow kinetics observed in *rd/rd cl* mice are in part a consequence of retinal degeneration. The difference in firing rate at the two wavelengths was near maximal within 2 s of stimulus onset and was then maintained throughout presentation. It seems thus that a melanopsin contribution to the firing rate of these cells appears within 100–200 ms of light on, builds up over the subsequent <2 s (to compensate for a decay in outer retinal signals), and is then maintained without noticeable reduction for at least 60 s of steady illumination. Taken together these data suggest that (1) melanopsin influence extends to all LGN neurons with a “sustained” phenotype and (2) that its input substantially enhances their ability to maintain a relatively stable firing rate under extended light exposure.

### Visual Responses Are Deficient in *Opn4* ^−*/*−^ Mice

As a direct test of the hypothesis that melanopsin contributes to sustained activation in the mouse LGN we examined responses of melanopsin knockout (*Opn4* ^−*/*−^) mice to blue light stimulation. In these mice rod/cone function remains intact and mRGCs are present but lack intrinsic photosensitivity. Multiunit recordings revealed light responses throughout the visual thalamus in this genotype (Figure S6). Strikingly, however, the sustained component of these multiunit responses was substantially reduced compared to those of *Opn1mw^R^* or *rd/rd cl* animals.

**Figure 5:**
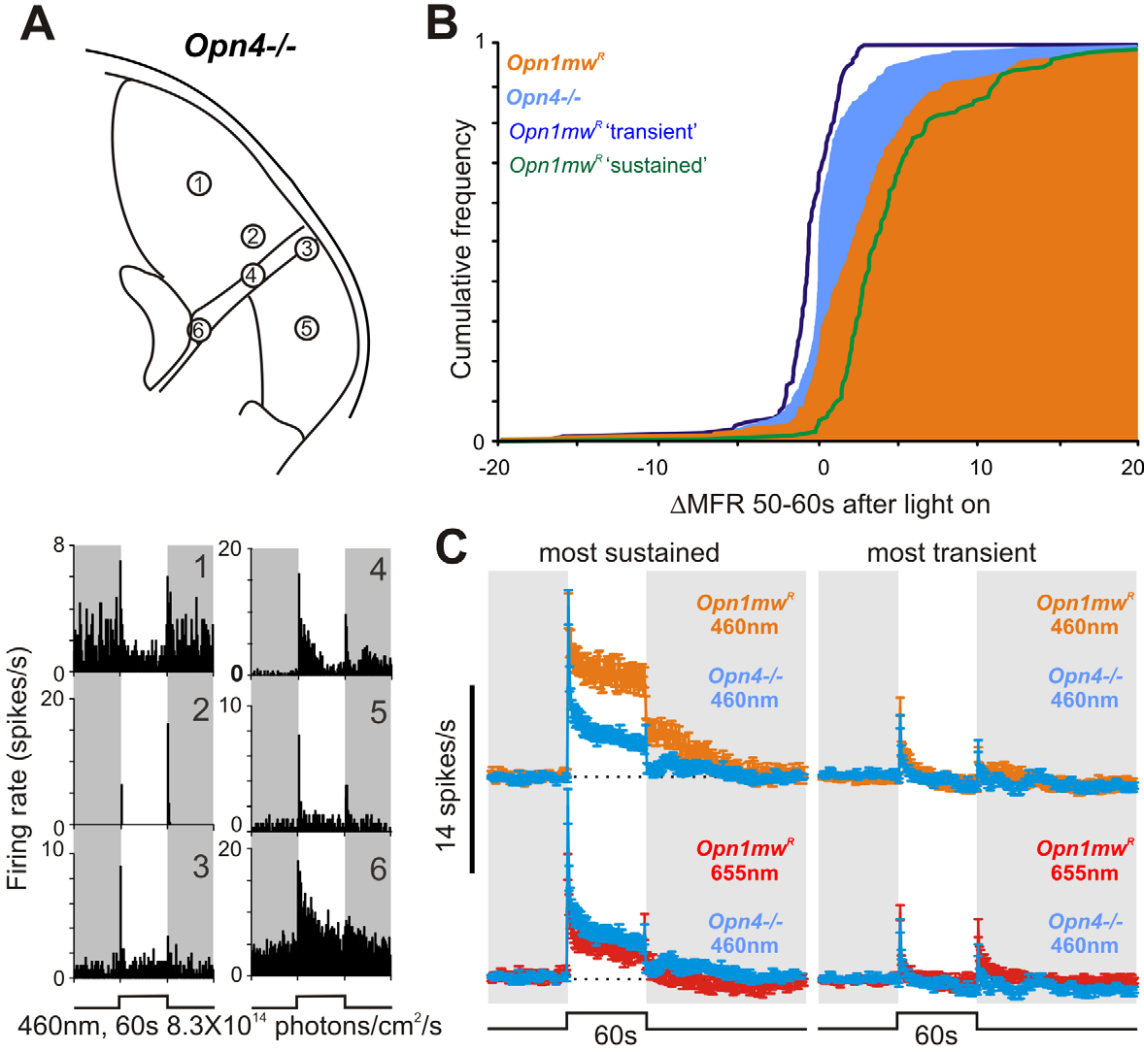
Sustained responses are deficient in melanopsin knockout mice. (A) Responses of six LGN cells from a representative melanopsin knockout mouse (*Opn4* ^−*/*−^; multiunit data in Figure S6), showing predominantly transient activations. (B) Cumulative frequency distribution of responses to 460 nm stimuli (8.3×10^14^ photons/cm^2^/s) for all light responses in *Opn4* ^−*/*−^ and *Opn1mw^R^* (solid areas) and separate “sustained” and “transient” subpopulations (lines) in *Opn1mw^R^*, quantified as the change in mean firing rate from baseline during the last 10 s of the light pulse. *Opn1mw^R^* was significantly different to *Opn4* ^−*/*−^, while *Opn1mw^R^* “transient” and *Opn4* ^−*/*−^ were not significantly different (Kolmogorov-Smirnov tests; *p*<0.001 and *p*>0.05, respectively). (C) Left panel: The response (mean ± SEM) of the 40% of *Opn4* ^−*/*−^ cells with the most sustained light responses was deficient at 460 nm (top) but not 655 nm (bottom) compared to the equivalent population in *Opn1mw^R^* mice. Right panel: The remaining 60% of cells in these two genotypes showed equivalent responses (mean ± SEM) at both wavelengths.

The implication that the activity of “sustained” neurons in the LGN is impaired in this genotype was confirmed by analysis of single unit responses. 217 of 520 single units (from 10 mice) extracted from these records were light responsive. However, none showed the very high amplitude sustained responses found so widely in the other genotypes. This finding is apparent in cumulative frequency plots for the change over baseline firing rate during the last 10 s of bright light exposure (Figure 5B). These revealed a significant difference between *Opn4* ^−*/*−^ and *Opn1mw^R^* genotypes (Kolmogorov-Smirnov test, D = 0.38, *p*<0.001), with the top 38% most “sustained” responses in *Opn1mw^R^* mice unaccounted for in the *Opn4* ^−*/*−^ distribution. The similarity between this figure and the proportion of “sustained” neurons in *Opn1mw^R^* mice (46%) and light responsive cells in the *rd/rd cl* LGN (∼35%) supports the view that ∼40% of light responsive neurons in the mouse LGN receive melanopsin input.

Although “sustained” responses across the *Opn4* ^−*/*−^ LGN were markedly deficient (Figure S7), they were not entirely absent. Thus, if analysis was restricted to the 40% of *Opn4* ^−*/*−^ cells with the most sustained responses (greatest increase in firing rate over baseline in the last 10 s of light exposure, Figure 5C) firing was elevated throughout 60 s of exposure. Indeed, while their response was significantly reduced compared to that of the *Opn1mw^R^* “sustained” population at 460 nm, it was equivalent to responses of those cells to “melanopsin-silent,” 655 nm, stimuli (mean ± SEM increases over baseline 0–60 s after light on; 3.5±0.5, 8.0±0.7, and 2.5±0.5 spikes/s, respectively; one-way ANOVA with Bonferroni post-test, *p*<0.0015 and *p*>0.05, respectively). In contrast, the 60% of *Opn4* ^−*/*−^ cells with the most transient responses were indistinguishable from those of the *Opn1mw^R^* “transient” population at either wavelength (Figure 5C; mean ± SEM increases over baseline 0–2 s after light on; 2.3±0.4, 3.1±0.6, and 2.5±0.4 spikes/s, respectively; one-way ANOVA with Bonferroni post-test, all *p*>0.05). These data therefore indicate that the outer retinal contribution to both “sustained” and “transient” responses is largely intact in *Opn4* ^−*/*−^ mice but that melanopsin loss is associated with a selective reduction in the amplitude of sustained activation.

### Melanopsin Contributions to Coding Irradiance

**Figure 6:**
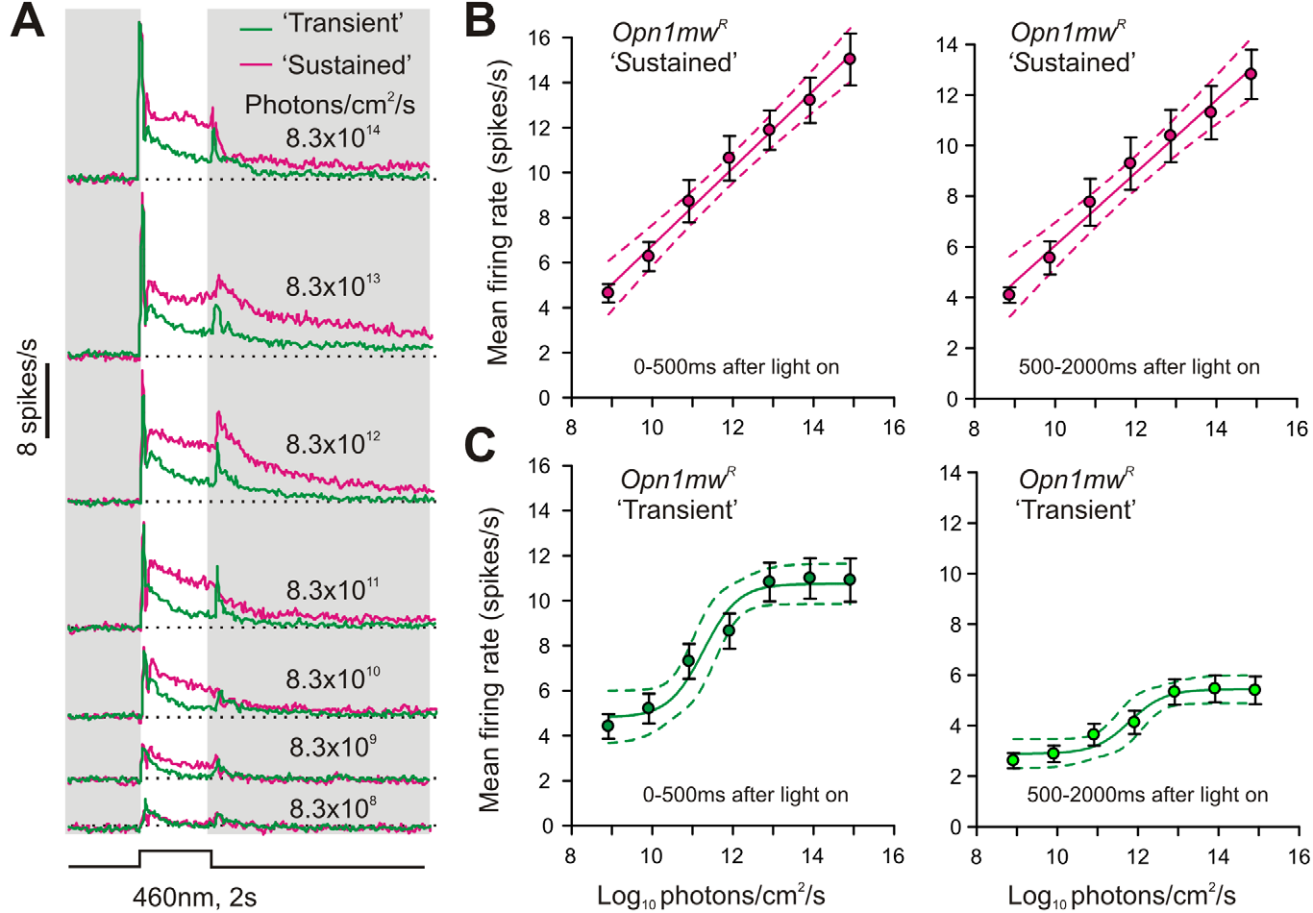
Irradiance coding by “sustained” lateral geniculate neurons. (A) Mean response of “transient” and “sustained” *Opn1mw^R^* LGN neurons (*n* = 134 and 114, respectively) to 2 s blue light pulses. (B,C) Quantification of the firing rate of *Opn1mw^R^* “sustained” (B) and “transient” (C) LGN cells during the first 500 ms (left) or remainder (500–2000 ms; right) of the light pulse. Symbols indicate mean (± SEM), and lines indicate mean (±95% CI) of the function that best described the data. Note the linear relationship between firing rate and log irradiance across the full range tested for *Opn1mw^R^* “sustained” cells in contrast to the sigmoidal relationship saturating above 10^12^ photons/cm^2^/s for *Opn1mw^R^* “transient” responses.

The LGN's reliance upon melanopsin to sustain its response to steady light exposure reveals that mRGCs make a unique contribution to visual physiology. To explore this contribution in more detail, we described full irradiance response relationships for “sustained” and “transient” units in the *Opn1mw^R^* LGN (Figure 6). We found that “sustained” firing had a remarkable ability to encode stimulus intensity. Thus, “sustained” cells showed a monotonic increase in firing rate over at least 6 decimal orders of stimulus irradiance, both in their initial phasic activation and in more tonic components of the response (Figure 6B). By contrast, responses of the “transient” population (Figure 6C) reached a plateau between 10^12^ and 10^13^ photons/cm^2^/s (around the predicted saturation point for mouse cones under these conditions [48] and the threshold for melanopsin-dependent responses in the *rd/rd cl* LGN; Figure 2E). Given the reliance of “sustained” firing on melanopsin, and consistent with our demonstration that melanopsin responses become apparent within 200 ms, these data suggest that mRGCs play an important role in encoding the intensity (irradiance) of our light step at both acute and sustained phases of presentation.

**Figure 7:**
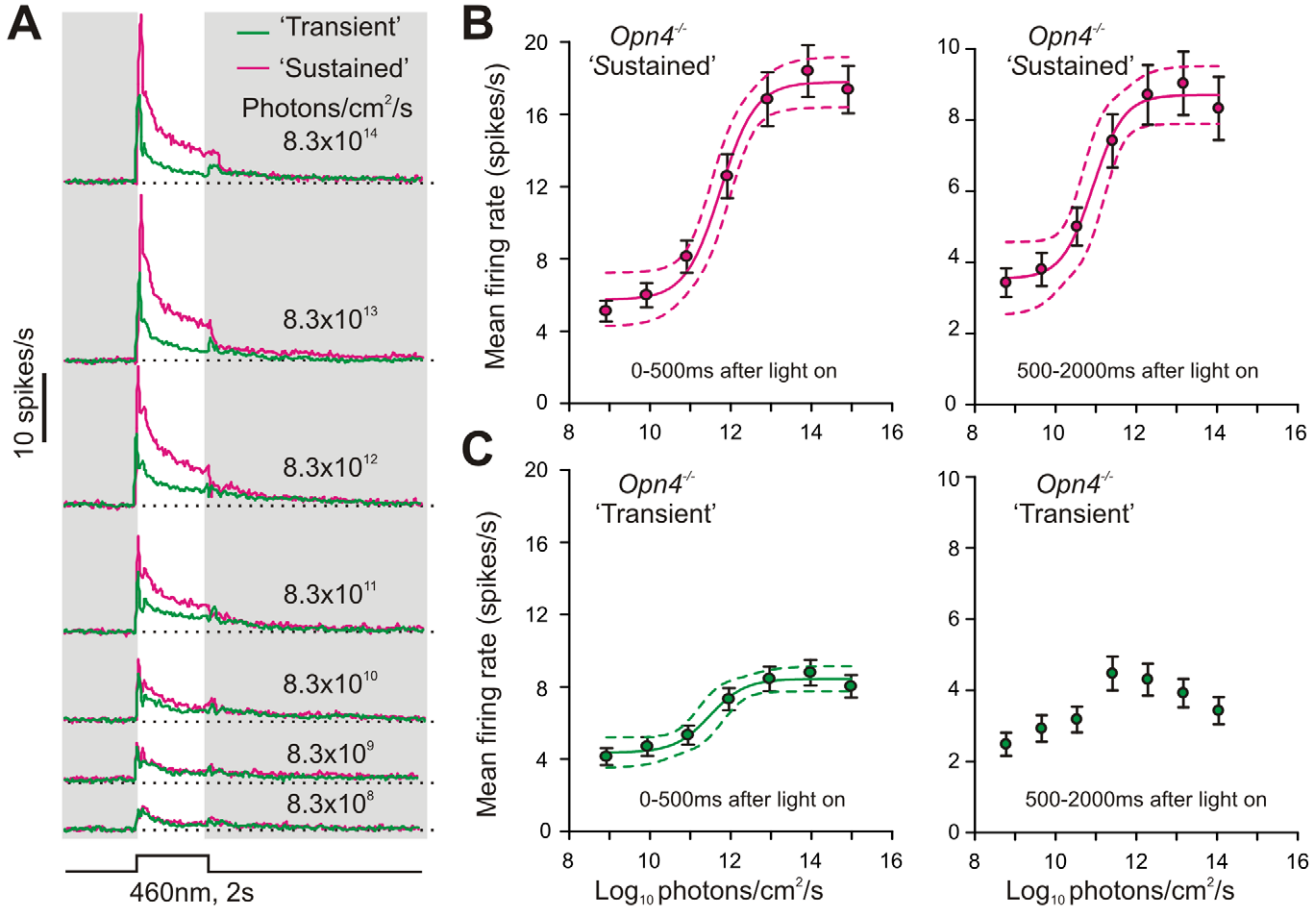
Melanopsin is necessary for encoding high irradiances. (A) Mean response to 2 s blue light pulses from the 40% most “sustained” and remainder 60% most “transient” *Opn4* ^−*/*−^ LGN neurons (as defined by their response to bright 60 s light steps; *n* = 87 and 130, respectively). (B,C) Quantification of the firing rate of *Opn4* ^−*/*−^ “sustained” (B) or “transient” (C) LGN cells during the first 500 ms (left) or remainder (500–2000 ms; right) of the light pulse. Symbols indicate mean ± SEM, and lines indicate mean ±95% CI of the function that best described the data. Note that, unlike *Opn1mw^R^* “sustained” cells, both “sustained” and “transient” *Opn4* ^−*/*−^ populations display sigmoidal relationships between firing rate and irradiance, which saturate above 10^12^ photons/cm^2^/s. Data for “transient” *Opn4* ^−*/*−^ cells over 500–2,000 ms of the response were not well fit by either linear or sigmoidal functions.

Melanopsin's contribution to such irradiance information was finally confirmed in *Opn4* ^−*/*−^ mice. Hence, in contrast to the extremely wide dynamic range of *Opn1mw^R^* “sustained” cells, responses of the equivalent “sustained” population in the *Opn4* ^−*/*−^LGN (as defined above) saturated above 10^12^ photons/cm^2^/s (Figure 7). This was not an artifact of the relative difficulty in identifying “sustained” cells in *Opn4* ^−*/*−^ mice, because even when the distinction between sustained and transient cells was ignored and the total LGN response assessed, irradiance coding was markedly deficient in the *Opn4* ^−*/*−^ compared to *Opn1mw^R^* LGN (Figure S8). These data indicate therefore that *Opn4* ^−*/*−^ LGN neurons entirely lack the ability to encode irradiances >10^13^ photons/cm^2^/s. Thus it seems that mechanisms of irradiance coding for central visual pathways are similar to those previously reported for the pupillomotor system [42],[50],[51], with rod/cone pathways encoding dim to moderate irradiances (<10^12^ photons/cm^2^/s), and melanopsin taking over at higher light levels

## Discussion

Using anatomical and physiological experiments we provide here a direct examination of melanopsin contributions to the activity of the thalamo-cortical visual pathway. We find that these rare photoreceptors (<2,000 per mouse retina compared with many hundreds of thousands of rods and cones) make a disproportionate contribution to visual activity. Thus, our experiments reveal that melanopsin sets the tonic firing rate under steady illumination of around 40% of all light responsive units in the LGN. As mRGCs also provide the major source of light information for accessory visual centers in the hypothalamus and pretectum [6],[7],[11], it seems that, despite their small numbers, these are among the most influential of all ganglion cell classes.

Our data suggest that melanopsin contributes a quality of visual information to the thalmo-cortical pathway that is unavailable from rods or cones. The experiments in mice with intact retinas (*Opn1mw^R^*) reveal that the “sustained” class of LGN neuron can encode irradiance over a very wide sensitivity range (at least 6 decimal orders). However, this ability is impaired in *Opn4* ^−*/*−^ mice, which can track irradiance only over a narrow range of dim-moderate intensities. The *Opn4* ^−*/*−^ LGN shows response saturation at around the threshold for melanopsin phototransduction (∼10^12^ photons/cm^2^/s). These data therefore indicate an irradiance dependent switch from rod/cone to melanopsin phases of irradiance coding in the LGN that is reminiscent of the situation for other melanopsin-influenced responses [4],[42],[50],[51]. In this regard, our data support the view that melanopsin photoreceptors compensate for a fundamental limitation in the ability of cones to encode irradiance over the photopic range. This may be explained by saturation in the “steady-state” level of cone hyperpolarization under extended illumination [52],[53]. Under these conditions photoreceptor adaptation and bleaching desensitization should allow cones to track high frequency modulations in light intensity, without necessarily encoding longer-term changes in illumination. Our data suggest that the visual system relies upon melanopsin to provide this latter information.

Insofar as melanopsin enhances the visual system's ability to measure light intensity, our data raise the possibility of an mRGC contribution to any aspect of mammalian vision that requires an accurate assessment of luminance/illuminance. Indeed, the finding that melanopsin influences essentially all thalamic neurons capable of luminance detection (the sustained population) in the mouse represents strong evidence for such a wide-ranging role in that species. If this were true also of humans, then mRGCs may contribute to as yet poorly understood mechanisms of “brightness” and “lightness” perception [54],[55]. For the moment, however, the aspect of human vision most directly addressed by our mouse experiments is spatial brightness perception. Just like our mouse thalamic neurons, human subjects can accurately estimate full field illumination over at least 8 decimal orders [56]. Based on our findings, we predict that melanopsin is responsible for at least the top 3–4 orders of this sensitivity range. In support of this possibility are reports that other features of human brightness perception seem inconsistent with cone physiology [57] and that high color temperature (“bluer”) lights appear brighter to human observers even when matched for photopic luminance [58]–[60].

An important unanswered question is whether mRGC input to the dLGN adopts a retinotopic order allowing it to encode spatial information. The resolution of any such mRGC-dependent spatial map would be low. Thus, if we assume a 90° field of view in mice with a fully constricted pupil and tiling of ∼28×28 for those mRGCs projecting to the dLGN (at most half of the total population of mRGCs), then acuity would be ∼0.3 cycles/° at best. Behavioral and electrophysiological experiments suggest that mouse visual acuity is not much superior to this [44],[61]–[63]. However, given their anatomy, and the kinetics of melanopsin phototransduction, it is hard to imagine that the selective advantage of retaining mRGC input to the dLGN lies in their ability to provide fine spatial information. It seems much more likely that if mRGCs do provide spatial information it takes the form of a relatively crude map of “brightness” to be used alongside higher acuity information in downstream visual processing.

The discovery of widespread melanopsin input to the thalamo-cortical visual pathway could have special relevance for the blind. Millions worldwide suffer varying degrees of blindness thanks to rod/cone degeneration or dysfunction. mRGCs survive even complete rod and cone loss and can support reflex light responses under these conditions. Our demonstration of extensive light driven activation in the LGN and cortex of *rd/rd cl* mice (an animal model of advanced retinal degeneration) raises the possibility that mRGCs also contribute to aspects of visual perception in these individuals [64]. Crude light perception typically survives in patients with retinal degeneration and there has been a suggestion that this could originate with melanopsin [10],[65] (although note that not all subjects with circadian light responses, and thus functional melanopsin photoreception, retain conscious light perception [66]).

If surviving melanopsin photoreceptors might support simple light perception in those with retinal degeneration, could they also provide spatial information? Very recently, Ecker and coworkers reported that *Gnat1* ^−*/*−^ *;Cnga3* ^−*/*−^ mice can use visual gratings to navigate a maze [18]. Insofar as these animals are considered to lack critical components of rod and cone phototransduction cascades (but have structurally intact retinae), that finding argues that mRGCs alone can support pattern vision. However, our work in *rd/rd cl* mice raises doubt over whether this could occur in advanced retinal degeneration. Thus, while melanopsin drives thalamic responses within 200 ms of a change in illumination in mice with an intact retina, responses build up very gradually in *rd/rd cl* animals, being first apparent at around 2 s after lights on, increasing over subsequent 10 s of seconds, and decaying very slowly at lights off. These kinetics seem incompatible with timeframes of visual fixation and would therefore probably preclude this melanopsin photoreception from providing useful spatial information. In agreement with this prediction, the ability to perceive light typically outlasts spatial discrimination in patients with progressive retinal dystrophy.

The discovery of photoreceptive retinal ganglion cells has thus far had little impact on our understanding of the processes of pattern vision and perception. We show here that these mRGCs are in fact influential regulators of electrophysiological activity in the primary visual pathway. Our data suggest that they are responsible for setting the tonic firing rate of 40% of thalamic neurons according to the level of illumination. This unique contribution implicates melanopsin in aspects of spatial brightness perception that have been hard to reconcile with the known capabilities of rods and cones [57]. The fact that these melanopsin signals appear in essentially all LGN neurons with a “sustained” response phenotype further identifies this inner retinal photoreceptor as a potential player in any visual process employing luminance information. These new functions add to the weight of evidence that good practice in architectural lighting should be revised to take account of the specific requirements of melanopsin photoreception.

## Methods

### Animals

All animal care was in accordance with local requirements, with studies in the United Kingdom conforming to the Animals, Scientific Procedures Act of 1986, and those at the Salk Institute approved by the Institutional Animal Care and Use Committee. Animals were kept in a 12-h dark/light cycle environment at a temperature of 22°C with food and water ad libitum.

### Alkaline Phosphatase (AP) Staining

The generation and genotyping of *Opn4^Cre/+^* mouse was described previously [11]. *Z/AP* mouse strain was purchased from Jackson Laboratory and was genotyped by X-gal staining on small biopsy samples following standard protocol [19]. AP staining of flatmount retina, retina sections, and of brain sections were done following protocols outlined in [67] with some modifications briefly described here. 8–12-wk-old *Opn4^Cre/+^;Z/AP* mice were deeply anesthetized with ketamine (70 mg/kg) and xylazine (10 mg/kg) and transcardially perfused with 4% paraformaldehyde. Retinas were postfixed for 15 min and washed twice in PBS with 2 mM MgCl_2_. Four small cuts were made on the retina to allow it to open for staining and imaging. Coronal brain sections (150 µm thickness) were cut into PBS by a vibratome and collected serially. Brain sections and retinas were heated at 65°C for 1 h in PBS to inactivate the endogenous alkaline phosphatase. Tissues were submerged in AP stain (0.1 M Tris-HCl, pH 9.5, 0.1 M NaCl, 50 mM MgCl_2_, 340 µg/ml NBT [nitroblue tetrazolium salt] and 175 µg/ml BCIP [5-bromo-4-chloro-3-indolyl phosphate, toluidinium salt] [NBT/BCIP kit, Cat# N-6547 from Invitrogen Carlsbad, CA]). AP staining was carried out for 30 min to overnight at room temperature with mild agitation. After staining, tissues were washed three times for 20 min each in PBS with 0.1% Tween 20. Brain sections and retinas were then postfixed overnight and 30 min, respectively, in 4% paraformaldehyde with Sorenson's Phosphate Buffer. Tissues were dehydrated through an ethanol series and cleared with 2∶1 benzyl benzoate:benzyl alcohol. Finally, tissues were mounted and visualized under Leica DC500 or Zeiss Stemi SV11. The number of cells per retina was counted with NIH Image J. A total of five *Opn4^Cre/+^;Z/AP* adult mice were bilaterally (3 mice) or unilaterally (2 mice) enucleated under ketamine/xylazine anesthesia. After 2 wk, tissues were subjected to AP staining procedure (see above).

### Immunostaining

Double staining for melanopsin and GFP in the retina flatmounts of *Opn4^Cre/+^;Z/EG* mice was carried out as described [11] with the following changes. Polyclonal rabbit anti-melanopsin antibodies raised against a synthetic peptide containing the N-terminus 15 amino acid of mouse melanopsin [68] was purified using protein-A magnetic beads following standard protocol. This purification step enhanced sensitivity and specificity of the antibody beyond published results. The purified antibodies at 1∶5000 dilution was used for immunostaining. For detection of GFP, a polyclonal chicken anti-GFP antibody was used at 1∶1000 dilution. FITC conjugated donkey anti-chicken IgG (1∶250, 703-095-155, Jackson ImmunoResearch) and Cy3 conjugated donkey anti-rabbit IgG (1∶500, 711-165-152, Jackson ImmunoResearch) were used for visualization.

### In Vivo Neurophysiology

#### Surgical procedures

Adult male mice (80–160 d) were anaesthetized by i.p. injection of 30% (w/v) urethane (1.7 g/kg; Sigma, Dorset, UK) and placed in a stereotaxic apparatus (SR-15M; Narishige International Ltd., London, UK). Additional top up doses of anesthetic (0.2 g/kg) were applied as required. Throughout the experiment the animal's temperature was maintained at 37°C with a homoeothermic blanket (Harvard Apparatus, Kent, UK). The skull surface was exposed and a small hole (∼1 mm diam.) drilled 2.5 mm posterior and 2.3 mm lateral to the bregma. The pupil, contralateral to the craniotomy, was dilated with topical application of 1% (w/v) atropine sulphate (Sigma) and the cornea kept moist with mineral oil. A recording probe (A4X8-5 mm-50-200-413; Neuronexus, MI, USA) consisting of four shanks (spaced 200 µm), each with eight recordings sites (spaced 50 µm), was then positioned centrally on the exposed skull surface, perpendicular to the midline, and lowered to a depth of 2.4–3.4 mm using a fluid filled micromanipulator (MO-10; Narishige). In most experiments, after recording from one location for 4–5 h the probe was raised or lowered 400 µm and a second set of responses recorded, such that overall the recording sites spanned a 4×16 grid 600 µm on the medial-lateral axis and 750 µm on the dorsal-ventral axis.

#### Recording methodology

Once the recording probe was in position the recording chamber was covered with darkroom blackout material (Nova Darkroom, Warwick, UK) and the room lights dimmed. Under these conditions background photon flux within the recording chamber was below the limit of our detectors. Mice were dark adapted for 1 h, which also allowed neuronal activity to stabilize after probe insertion/repositioning, after which we began recording. Neural signals were acquired using a Recorder64 system (Plexon, TX, USA). Signals were amplified ×3,000, highpass filtered at 300 Hz, and digitized at 40 kHz. Multiunit activity (spikes with amplitudes >50 µV) were saved as time stamped waveforms and analyzed offline (see below). Light stimuli were delivered by a custom built LED based light source (Cairn Research Ltd, Kent, UK), which consisted of independently controlled red and blue LEDs (λmax: 460 and 655 nm, respectively) with appropriate bandpass filters (half peak width: ±10 nm). The light passed through a filter wheel with various neutral density filters and was focused onto a 5 mm diameter piece of opal diffusing glass (Edmund Optics Inc., York, UK) positioned 3 mm from the eye contralateral to the recording probe. LED intensity and filter wheel position were controlled by a PC running LabView 8.6 (National instruments). Blue/red light stimulations were either applied for 60 s with an interstimulus interval of 540 s or for 2 s with an interstimulus interval of 38 s. At each intensity used (starting at the lowest) 3–5 trials (for 60 s pulses) or 20 trials (for 2 s pulses) were conducted before increasing the intensity. Mice were otherwise kept in complete darkness. Light measurements were performed using a calibrated spectrophotometer (Ocean Optics, FL, USA) and optical power meter (Macam Photometrics, Livingston, UK). Effective photon flux for each photoreceptor class was determined using the calculated spectra and visual pigment templates described by Govardovskii et al. [69].

#### Histological analysis

For each experiment the location of the probe was verified histologically. In most experiments, the electrode was immersed in fluorescent dye before recording (Cell Tracker CM-DiI; Invitrogen Ltd. Paisley, UK). In some of these experiments (and in all other experiments) at the end of recording lesioning, current pulses (35 µA, 6 s) were applied using a constant current stimulator (DS3, Digitimer Ltd., Herts., UK) to selected recording sites. In all cases, at the end of the experiment the mouse was perfused transcardially with 0.1 M phosphate buffered saline followed by 4% paraformaldehyde. The brain was removed, postfixed overnight, cryoprotected with 30% sucrose, then sectioned at 100 µm on a freezing sledge microtome. For detection of DiI fluorescence, sections were mounted with Vectashield (Vectorlaboratories Ltd. Peterborough, UK), coverslipped, and visualized using an Olympus BX51 with appropriate filter sets. When the fluorescent marker was not used, sections were Nissl stained. Sections were scaled to account for shrinkage using the distance between lesion sites and electrode tracks (for DiI fluorescence) and aligned with the corresponding mouse atlas sections [70] to estimate the stereotaxic coordinates of each recording site.

#### Data analysis

Multichannel, multiunit recordings were analyzed in Offline Sorter (Plexon). After removing artifacts we used principal component–based sorting to discriminate single units, identifiable as a distinct cluster of spikes in principal component space with a clear refractory period in their interspike interval distribution (Figure S9). In most cases we identified a single distinct unit at each recording site (total 2,113 units from 2,112 recording sites). Following spike sorting data were exported to Neuroexplorer (Nex Technologies, MA, USA) and MATLAB R2007a (The Mathworks Inc., MA, USA) for construction of peristimulus histograms and further analysis. Light responsive units were identified as those where the peristimulus average showed a clear peak (or trough) that exceeded the 99% confidence limits estimated from a Poisson distribution derived from the prestimulus spike counts.

### Retinal Tract Tracing

Male mice (80–160 d) were anaesthetized by i.p. injection of ketamine (70 mg/kg) and xylazine (7 mg/kg). Topical anesthetic (1% w/v amethocaine HCl; Chauvin Pharmaceuticals Ltd, Essex, UK) was applied to one eye and 2 µl of 1% cholera toxin β subunit conjugated to Alexa Fluor-488 (Invitrogen) was injected intravitreally using a glass needle (tip ∼20 µl) attached to a Hamilton syringe. Mice were allowed to recover for 3–4 d before perfusion and histological processing as described above for DiI fluorescence.

### Optical Imaging

#### Animal preparation

Adult (80–160 d) male *rd/rd cl* and appropriate wildtype control mice (C3H/f, wild type for the rd1 mutation) were anaesthetized as for in vivo neurophysiological recordings (above) and eye protection cream was applied to the left cornea. A tracheotomy was performed to reduce the incidence of breathing problems [71] and the skin above the skull retracted. A black plastic cylindrical chamber was fixed over the cortex using dental cement, filled with 37°C saline and a cover slip was placed on top. The right eye was occluded using a plastic black mask fixed using dental cement and the pupil of the left eye was dilated by topical application of 1% atropine sulphate. Eye protection cream was then reapplied and a mouse contact lens with no refractive correction was placed on it to protect the cornea.

#### Visual stimulation

The visual stimulus was delivered using a xenon-arc lamp and passed through a 447 nm broadband (60 nm bandwidth) filter (Edmund Optics) and delivered onto a 25 mm diffuser placed 10 mm from the left eye giving an irradiance of 2.9×10^14^ photons/cm^2^/s. The animal was dark adapted for 30 min before starting visual stimulation. The duration of the stimulus lasted for 20 s. For averaging purposes the stimulus was repeated 15–20 times with an inter-stimulus interval of 120 s. Subsequently, recordings were taken using a “blank stimulus” during which no visual stimulus was delivered; in no case did we observe any cortical response under these “blank stimulus” conditions.

#### Data collection

In order to perform single wavelength optical imaging the cortex was illuminated using a halogen lamp. Frames were taken from the cortex using a cooled 12 bit SMD (1M60) digital camera mounted on top of a Leica MZ75 microscope using a magnification of 1.6. Custom-made software was written using Visual Studio. NET to synchronize the camera recordings with the visual stimulus by triggering the opening of an electronic shutter (Melles-Griot, Cambridge, UK) placed in the light stimulus pathway. The cortex was first illuminated using an interference filter of 570 nm (Edmund Optics) to focus the camera on the surface vascular pattern. The camera was then focused 700 µm below the surface and a near-infrared filter of 692 nm (40 nm bandwidth) was used during data collection. A total of 80 s were recorded per stimulus repetition starting 20 s before stimulus onset. Frames were binned with a 4×4 spatial binning stored at 8 Hz and downsampled to 2 Hz before further analysis.

#### Data analysis

The data were averaged over stimulus repetitions on a frame by frame basis. Frames were averaged in 5 s intervals and subtracted from those frames taken within the last second before stimulus onset. In this way, maps of activity are produce by highlighting the spatial changes in the optical imaging signal following stimulus presentation. In order to average maps of activity across animals from each group, the cortical maps were aligned with respect to the bregma coordinate and then averaged for corresponding frames. An approximate location of the cortical regions imaged was estimated from coronal sections of published mouse stereotaxic coordinates [72].

## Supporting Information

Figure S1 **Genetic and immunohistochemical co-labeling of melanopsin retinal ganglion cells.** Representative retinal sections from *Opn4^Cre/+^;Z/EG* mice co-labeled with a purified rabbit polyclonal antibody raised against an N-terminus epitope of mouse melanopsin [68]. (A) Anti-Opn4-immunofluoresence (red), (B) GFP expression (green), (C) merge. We found 110–130 GFP positive cells/mm^2^, which amounts to ∼1,500 cells in the adult mouse retina (based on an area of 14 mm^2^). Of these cells 86.4% were double labeled (white arrows in A) and 10.2% were GFP positive (green arrows) but lacked detectable melanopsin immunostaining, presumably due to a very low level of melanopsin expression undetectable by the antibody. These GFP positive soma were all restricted to the ganglion cell layer and proximal zone of the inner nuclear layer. There were also some sparse cells (2–6/mm^2^; red arrows) staining only with anti-melanopsin antibody. In these cells GFP is not expressed to a detectable level owing to insufficient Cre function or GFP expression. The dendrites of all GFP or melanopsin immunospositive cells stratified almost equally in both proximal and distal zones of the inner plexiform layer (IPL). Since the M1 type of melanopsin cells primarily stratify in the distal zone of the IPL [11],[13]–[15], the Cre expressing retinal ganglion cells in mice mark both M1 and additional cell types expressing melanopsin.
(2.12 MB TIF)

Figure S2 **Anatomy of monocular projections of melanopsin ganglion cells.** Representative sections (150 µm thick) from unilaterally enucleated *Opn4^Cre/+^;Z/AP* mice stained with chromogenic alkaline phosphatase substrate. mRGC innervations to the (A,B) superior colliculus (SC), (C,D) olivery pretectal nuclei (OPN), and (E,F) lateral geniculate nucleus (LGN) are predominantly contralateral. As shown previously the (G) SCN receives bilateral innervation of mRGC from each retina.
(3.64 MB TIF)

Figure S3 **Comparative anatomy of monocular retinal projections in** ***rd/rd cl*** **and** ***Opn1mw^R^*** **mice.** Representative sections (100 µm thick) from *rd/rd cl* (left) and *Opn1mw^R^* (right) mice showing cholera toxin β subunit-Alexa Fluor 488 fluorescence across all major retinal targets. The density and ration of ipsilateral:contralateral retinal innervation was similar across all retinorecipient target sites and consistent with previous reports [13].
(0.99 MB TIF)

Figure S4 **Light response waveforms in thalamic sub-regions of** ***rd/rd cl*** **mice.** The average single unit response waveforms following 60 s, 460 nm, illumination in *rd/rd cl* mice were similar regardless of the projected anatomical location of the cell. Data show the mean ± SEM change in firing rate of light responsive cells detected in the dorsal LGN (top; *n* = 84), intergeniculate leaflet (IGL) and ventral LGN (middle; *n* = 221), or medial areas of the thalamus bordering the LGN (bottom; *n* = 39).
(0.31 MB TIF)

Figure S5 **Cone-independent sustained activation of lateral geniculate (LGN) neurons by blue light pulses.** (A,B) Multichannel, multiunit recordings from the LGN of a representative red cone knockin mouse (*Opn1mw^R^*) showing widespread and sustained neuronal activation in response to 460 nm light pulses (60 s; 8.3×10^14^ photons/cm^2^/s). (C) 655 nm light pulses (60 s; 2.6×10^15^ photons/cm^2^/s) isoluminant to the 460 nm stimuli for cones evoked much more transient changes in neuronal activity. Traces in (B) and (C) represent the change in multiunit firing (average of four responses) at corresponding recording sites (circles) in panel A; shaded areas represent interstimulus periods of darkness.
(0.58 MB TIF)

Figure S6 **The melanopsin knockout LGN lacks high amplitude sustained responses.** (A,B) Multichannel, multiunit recordings from the lateral geniculate (LGN) of a representative melanopsin knockout mouse (*Opn4* ^−*/*−^) showing predominantly transient on and off activations in response to 60 s light pulses (8.3×10^14^ photons/cm^2^/s at 460 nm). Traces in (B) represent the change in multiunit firing (average of three responses) at corresponding recording sites (circles) in panel A; shaded areas represent darkness. (C) Anatomical distribution of light responsive cells detected in all *Opn4* ^−*/*−^ mice investigated, relative to the total number of cells found in each 200 µm×200 µm grid square (based on 520 units recorded in 10 mice).
(0.22 MB TIF)

Figure S7 **Sustained responses are deficient in melanopsin knockout LGN neurons.** Average (± SEM) response of all *Opn4* ^−*/*−^ cells (*n* = 217) to 460 nm light compared with responses of all *Opn1mw^R^* neurons (i.e. “sustained” + “transient” subpopulations, *n* = 248) to 460 nm (top) and 655 nm (bottom). Responses of *Opn4* ^−*/*−^ neurons were significantly smaller than those of *Opn1mw^R^* cells at 460 nm but similar to those at 655 nm (mean ± SEM increases over baseline 0–60 s after light on; 1.3±0.3, 3.3±0.5, and 1.1±0.3 spikes/s, respectively; one-way ANOVA with Bonferroni post-test, *p*<0.001 and *p*>0.05, respectively).
(0.16 MB TIF)

Figure S8 **Irradiance coding is deficient in the lateral geniculate (LGN) of** ***Opn4*** **^−^** ^***/*−**^ **mice.** (A) Average response of all light activated LGN neurons recorded in *Opn4* ^−*/*−^ (blue; *n* = 217) and *Opn1mw^R^* mice (orange; including both “sustained” and “transient” populations; *n* = 248) to 2 s blue light pulses. (B,C) Quantification of the firing rate of all light responsive LGN cells in *Opn4* ^−*/*−^ (B) and *Opn1mw^R^* (C) mice during the first 500 ms (left) or remainder (500–2000 ms; right) of the light pulse. Symbols indicate mean (± SEM), and lines indicate mean (±95% CI) of the function that best described the response. Note that even though irradiance coding is a unique property of “sustained” neurons (Figure 6), a clear linear relationship between irradiance and firing rate is apparent in the pooled responses of “sustained” and “transient” cells in *Opn1mw^R^* mice. Thus, the deficiency of this activity in *Opn4* ^−*/*−^ mice (B and Figure 6) does not merely reflect our inability to separate “sustained” and “transient” cell types in this genotype.
(0.65 MB TIF)

Figure S9 **Single unit spike discrimination.** (A) Mean (± SD) waveform of spikes assigned to three single units (corresponding to units 1–3 in Figure 4). (B) Spike patterns of single units from 0.5 s before to 1.5 s after the start of a 460 nm light pulse (8.3×10^14^ photons/cm^2^/s). (C) Expanded view of spike traces in (B) at the points marked *. (D) Log interspike interval (ISI) histograms for units 1–3. Histograms show a sharp peak at ISIs between 3 and 5 ms corresponding to spikes fired in bursts and broader peaks at longer ISIs corresponding to epochs of tonic firing.
(0.26 MB TIF)

## Abbreviations

AP: alkaline phosphatase
dLGN: dorsal lateral geniculate nuclei
IGL: Intergeniculate leaflet
mRGC: melanopsin-expressing retinal ganglion cell
OPN: Olivary pretectal nuclei
RSD: retrosplenial dysgranular cortex
SC: superior colliculus
SCN: suprachiasmatic nuclei
