# Supplementary figures and images for: Melanopsin Contributions to Irradiance Coding in the Thalamo-Cortical Visual System
Source: PLoS Biol. 2010 Dec 7;8(12):e1000558. doi: 10.1371/journal.pbio.1000558 (PMC2998442; doi:10.1371/journal.pbio.1000558)

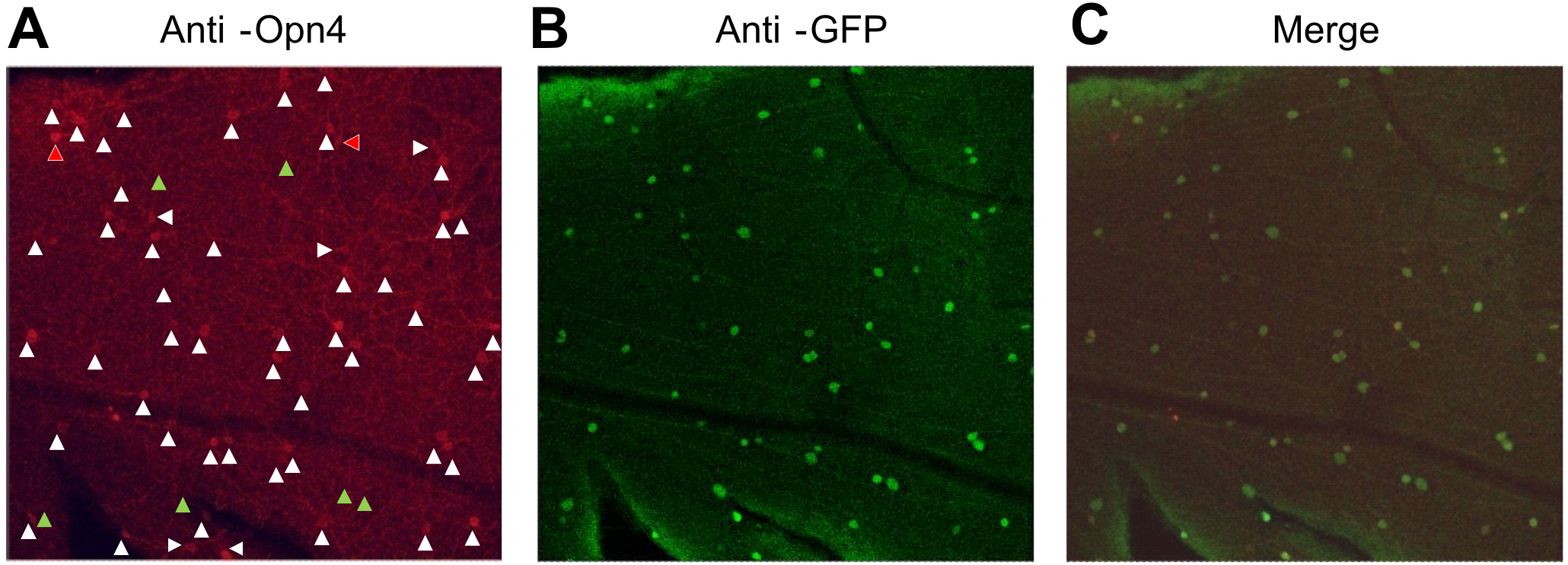

Supplement: Figure S1 — Genetic and immunohistochemical co-labeling of melanopsin retinal ganglion cells. Representative retinal sections from Opn4Cre/+;Z/EG mice co-labeled with a purified rabbit polyclonal antibody raised against an N-terminus epitope of mouse melanopsin [68]. (A) Anti-Opn4-immunofluoresence (red), (B) GFP expression (green), (C) merge. We found 110–130 GFP positive cells/mm2, which amounts to ∼1,500 cells in the adult mouse retina (based on an area of 14 mm2). Of these cells 86.4% were double labeled (white arrows in A) and 10.2% were GFP positive (green arrows) but lacked detectable melanopsin immunostaining, presumably due to a very low level of melanopsin expression undetectable by the antibody. These GFP positive soma were all restricted to the ganglion cell layer and proximal zone of the inner nuclear layer. There were also some sparse cells (2–6/mm2; red arrows) staining only with anti-melanopsin antibody. In these cells GFP is not expressed to a detectable level owing to insufficient Cre function or GFP expression. The dendrites of all GFP or melanopsin immunospositive cells stratified almost equally in both proximal and distal zones of the inner plexiform layer (IPL). Since the M1 type of melanopsin cells primarily stratify in the distal zone of the IPL [11],[13]–[15], the Cre expressing retinal ganglion cells in mice mark both M1 and additional cell types expressing melanopsin. (2.12 MB TIF) [file pbio.1000558.s001.tif]

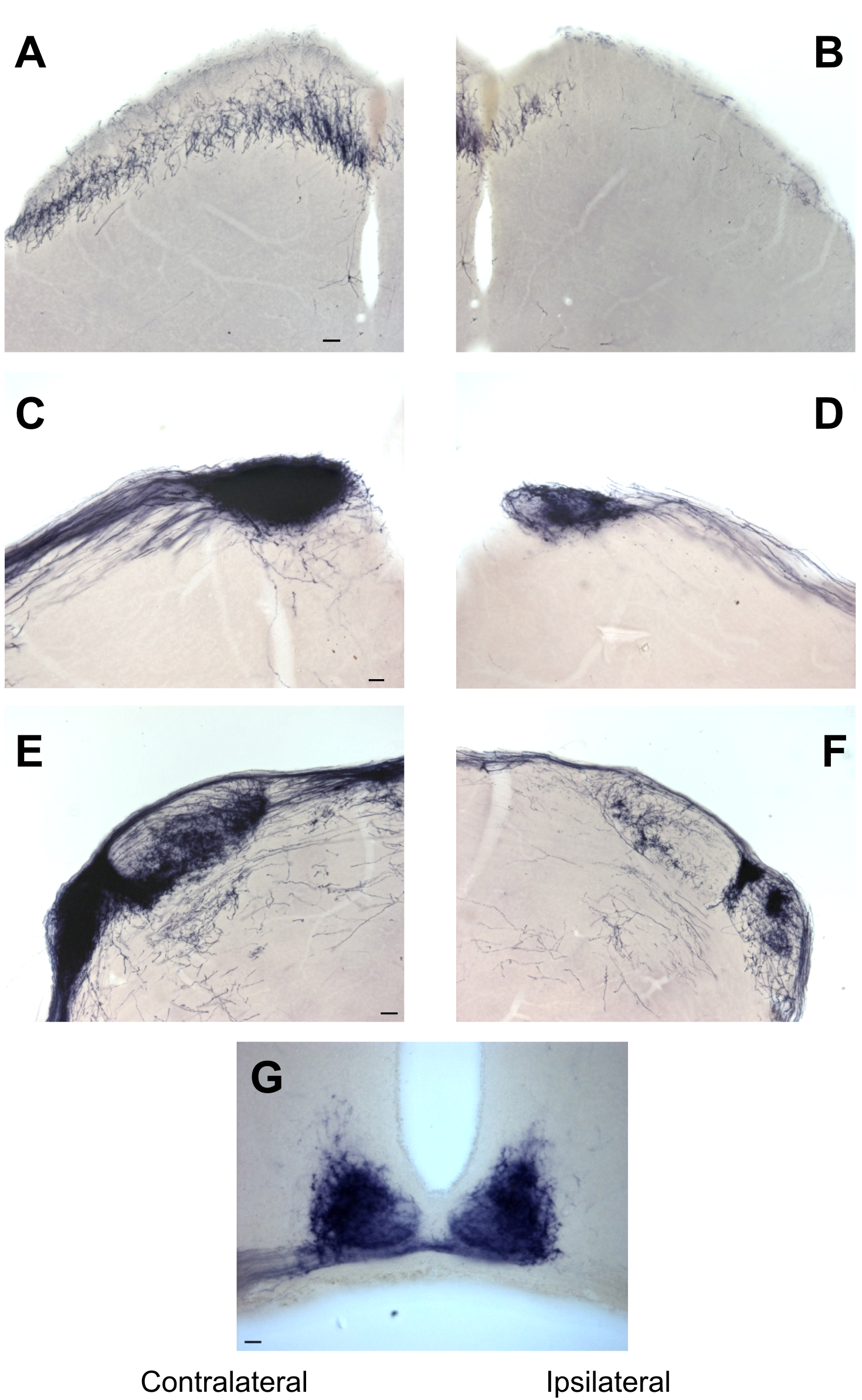

Supplement: Figure S2 — Anatomy of monocular projections of melanopsin ganglion cells. Representative sections (150 µm thick) from unilaterally enucleated Opn4Cre/+;Z/AP mice stained with chromogenic alkaline phosphatase substrate. mRGC innervations to the (A,B) superior colliculus (SC), (C,D) olivery pretectal nuclei (OPN), and (E,F) lateral geniculate nucleus (LGN) are predominantly contralateral. As shown previously the (G) SCN receives bilateral innervation of mRGC from each retina. (3.64 MB TIF) [file pbio.1000558.s002.tif]

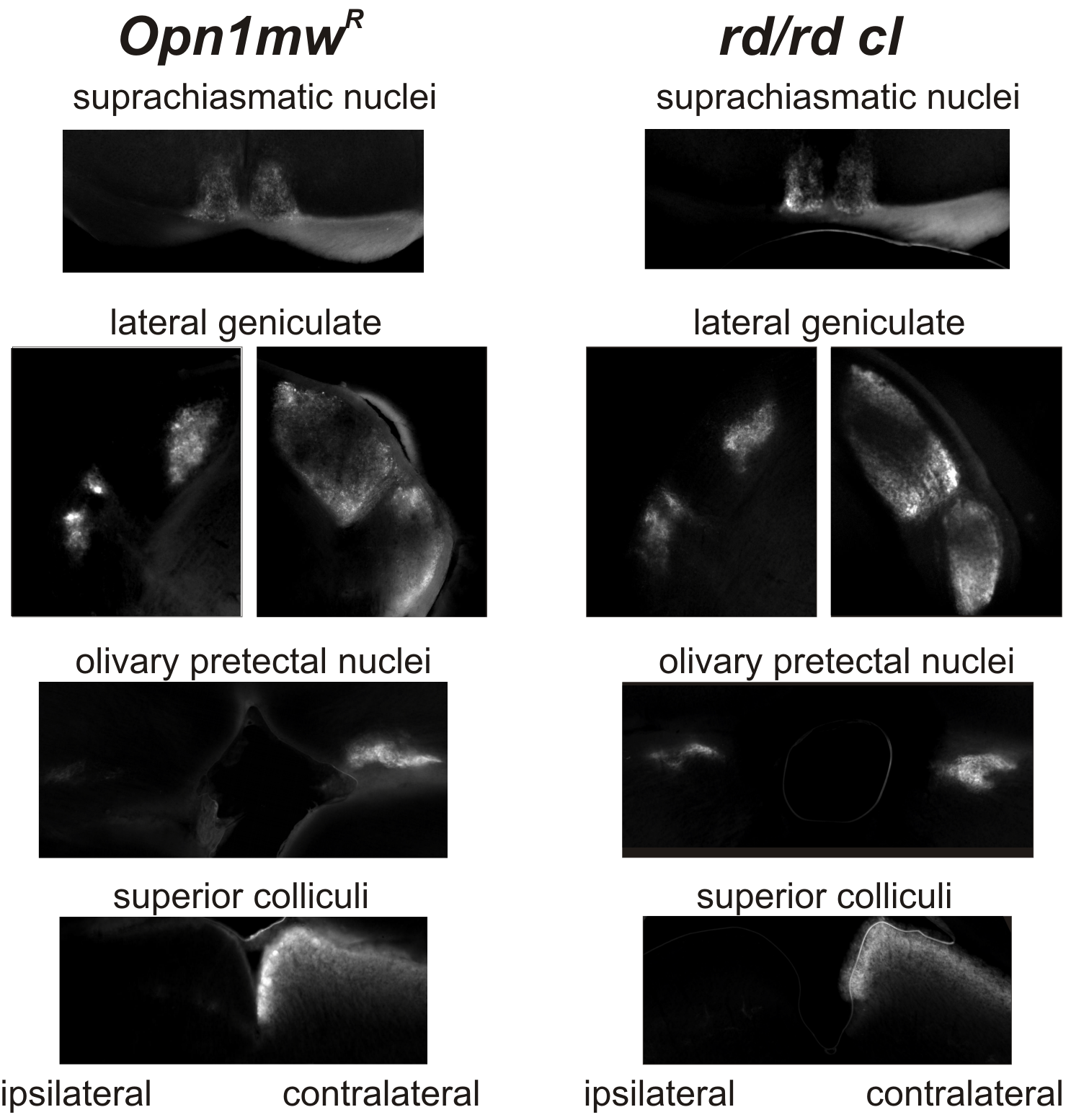

Supplement: Figure S3 — Comparative anatomy of monocular retinal projections in rd/rd cl and Opn1mwR mice. Representative sections (100 µm thick) from rd/rd cl (left) and Opn1mwR (right) mice showing cholera toxin β subunit-Alexa Fluor 488 fluorescence across all major retinal targets. The density and ration of ipsilateral:contralateral retinal innervation was similar across all retinorecipient target sites and consistent with previous reports [13]. (0.99 MB TIF) [file pbio.1000558.s003.tif]

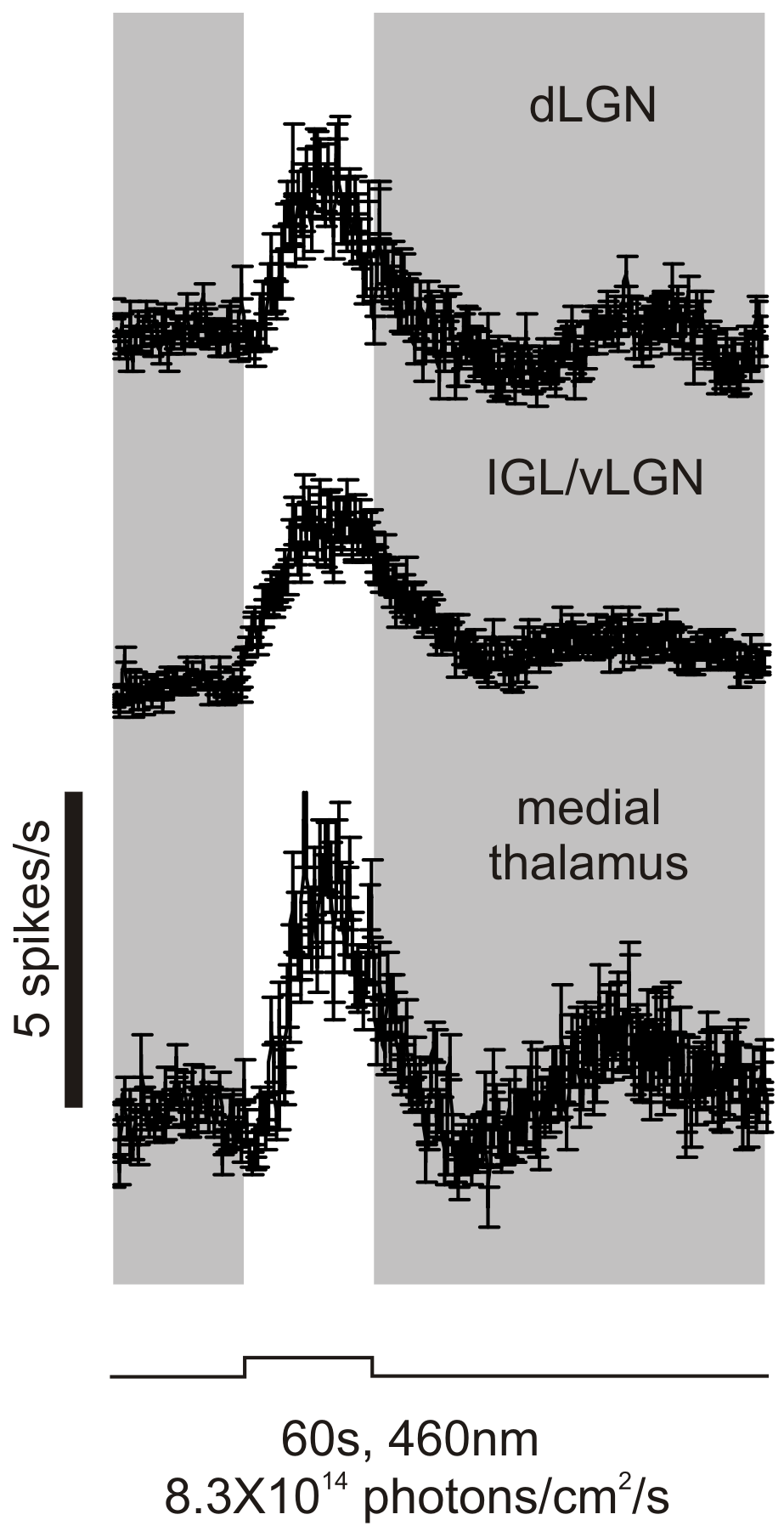

Supplement: Figure S4 — Light response waveforms in thalamic sub-regions of rd/rd cl mice. The average single unit response waveforms following 60 s, 460 nm, illumination in rd/rd cl mice were similar regardless of the projected anatomical location of the cell. Data show the mean ± SEM change in firing rate of light responsive cells detected in the dorsal LGN (top; n = 84), intergeniculate leaflet (IGL) and ventral LGN (middle; n = 221), or medial areas of the thalamus bordering the LGN (bottom; n = 39). (0.31 MB TIF) [file pbio.1000558.s004.tif]

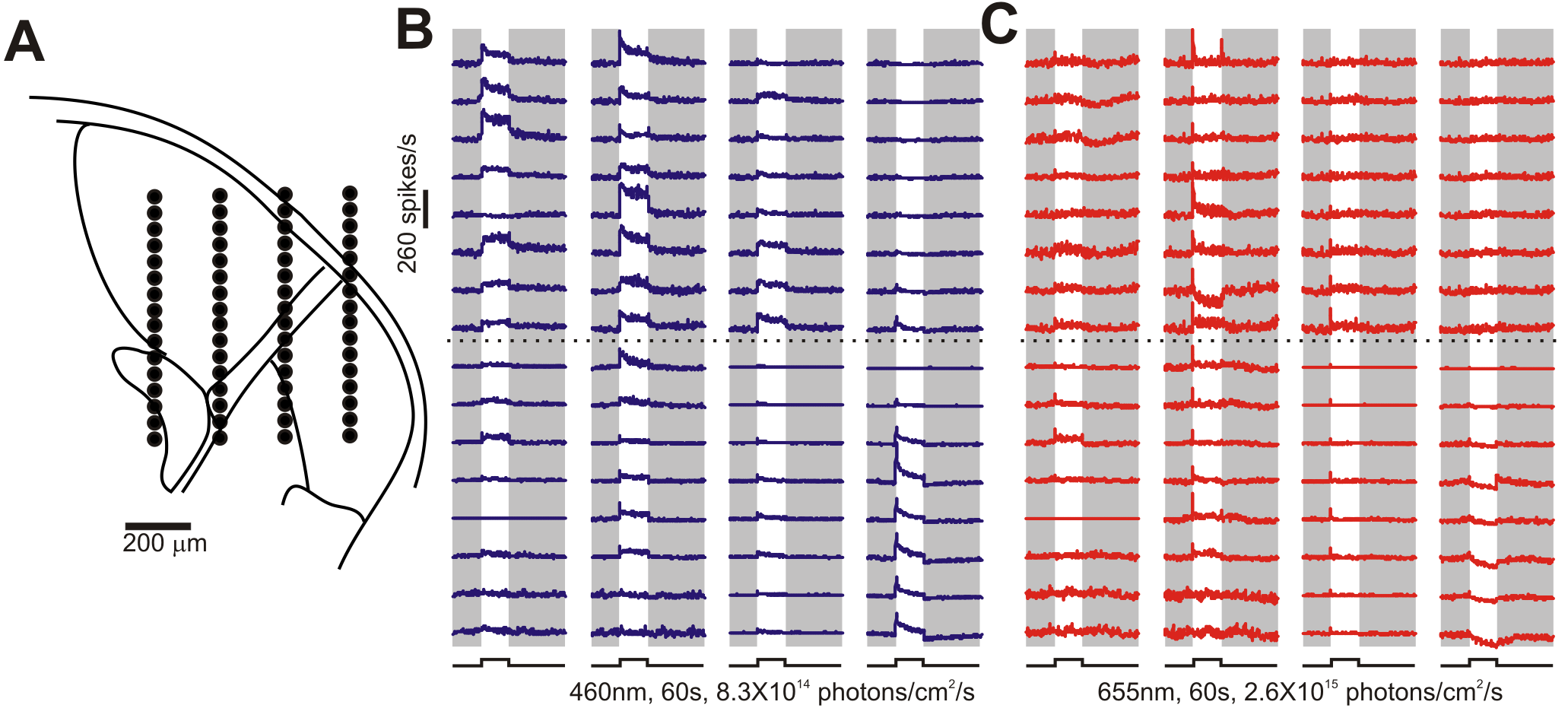

Supplement: Figure S5 — Cone-independent sustained activation of lateral geniculate (LGN) neurons by blue light pulses. (A,B) Multichannel, multiunit recordings from the LGN of a representative red cone knockin mouse (Opn1mwR) showing widespread and sustained neuronal activation in response to 460 nm light pulses (60 s; 8.3×10^14^ photons/cm2/s). (C) 655 nm light pulses (60 s; 2.6×10^15^ photons/cm2/s) isoluminant to the 460 nm stimuli for cones evoked much more transient changes in neuronal activity. Traces in (B) and (C) represent the change in multiunit firing (average of four responses) at corresponding recording sites (circles) in panel A; shaded areas represent interstimulus periods of darkness. (0.58 MB TIF) [file pbio.1000558.s005.tif]

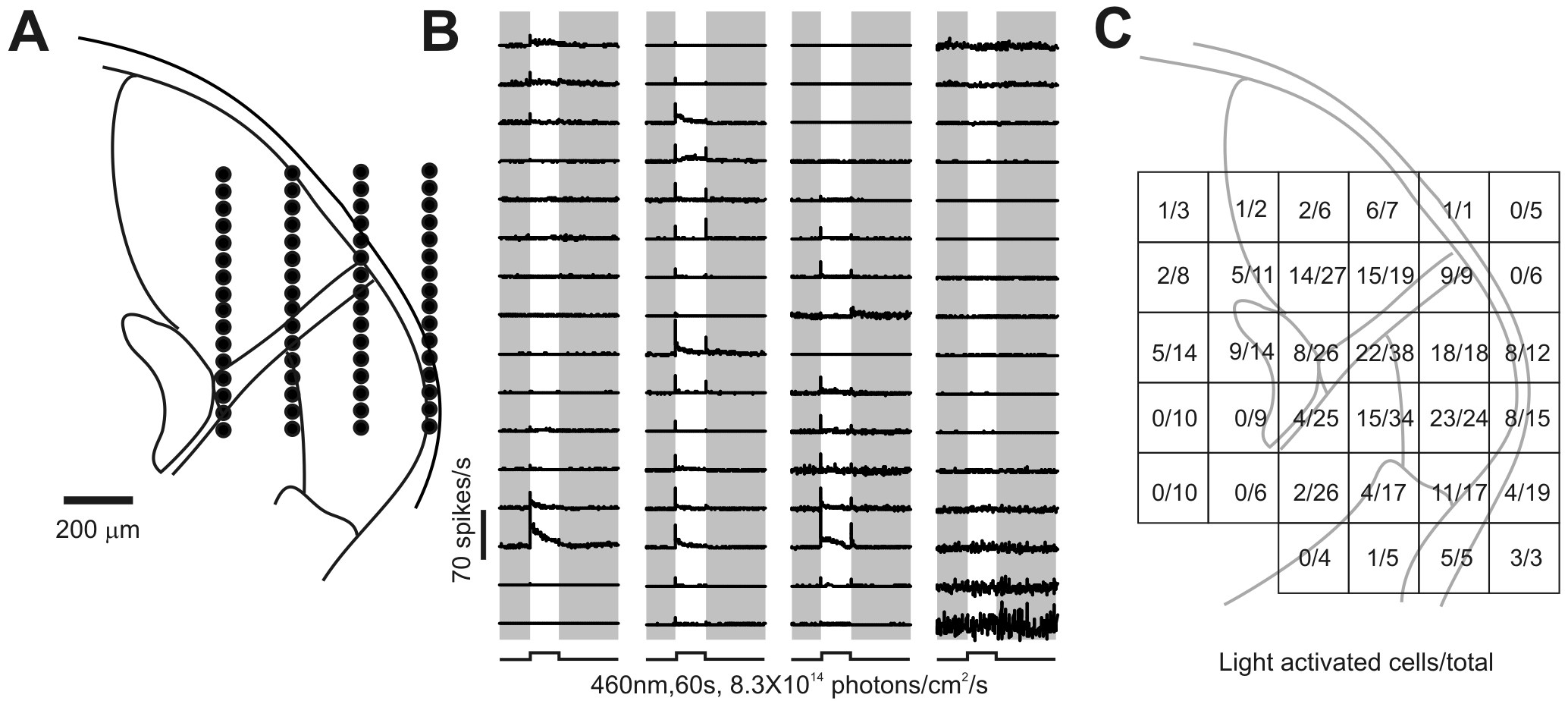

Supplement: Figure S6 — The melanopsin knockout LGN lacks high amplitude sustained responses. (A,B) Multichannel, multiunit recordings from the lateral geniculate (LGN) of a representative melanopsin knockout mouse (Opn4 −/−) showing predominantly transient on and off activations in response to 60 s light pulses (8.3×10^14^ photons/cm2/s at 460 nm). Traces in (B) represent the change in multiunit firing (average of three responses) at corresponding recording sites (circles) in panel A; shaded areas represent darkness. (C) Anatomical distribution of light responsive cells detected in all Opn4 −/− mice investigated, relative to the total number of cells found in each 200 µm×200 µm grid square (based on 520 units recorded in 10 mice). (0.22 MB TIF) [file pbio.1000558.s006.tif]

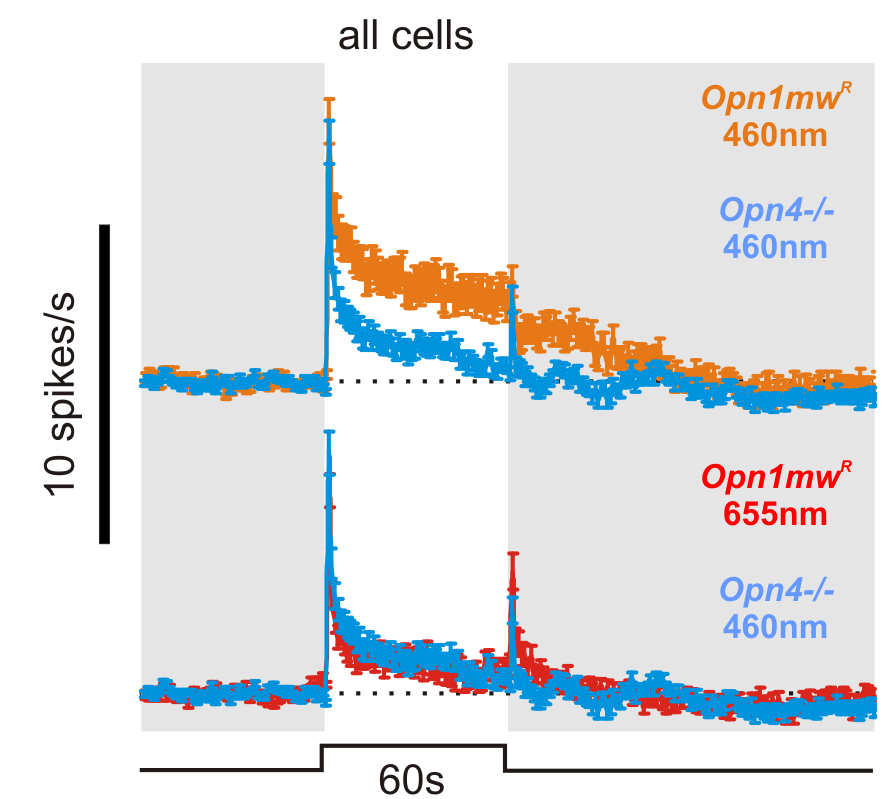

Supplement: Figure S7 — Sustained responses are deficient in melanopsin knockout LGN neurons. Average (± SEM) response of all Opn4 −/− cells (n = 217) to 460 nm light compared with responses of all Opn1mwR neurons (i.e. “sustained” + “transient” subpopulations, n = 248) to 460 nm (top) and 655 nm (bottom). Responses of Opn4 −/− neurons were significantly smaller than those of Opn1mwR cells at 460 nm but similar to those at 655 nm (mean ± SEM increases over baseline 0–60 s after light on; 1.3±0.3, 3.3±0.5, and 1.1±0.3 spikes/s, respectively; one-way ANOVA with Bonferroni post-test, p<0.001 and p>0.05, respectively). (0.16 MB TIF) [file pbio.1000558.s007.tif]

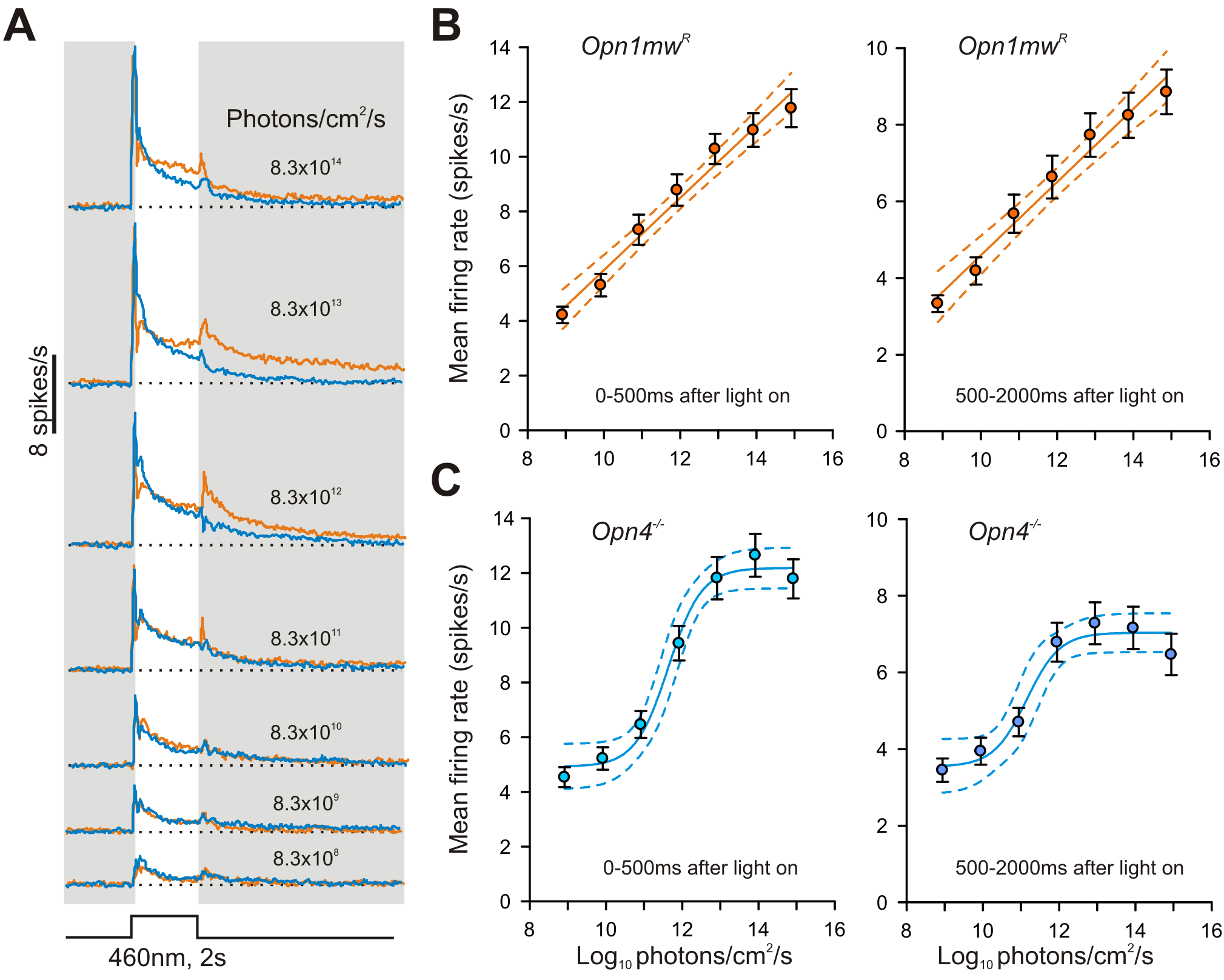

Supplement: Figure S8 — Irradiance coding is deficient in the lateral geniculate (LGN) of Opn4 − /− mice. (A) Average response of all light activated LGN neurons recorded in Opn4 −/− (blue; n = 217) and Opn1mwR mice (orange; including both “sustained” and “transient” populations; n = 248) to 2 s blue light pulses. (B,C) Quantification of the firing rate of all light responsive LGN cells in Opn4 −/− (B) and Opn1mwR (C) mice during the first 500 ms (left) or remainder (500–2000 ms; right) of the light pulse. Symbols indicate mean (± SEM), and lines indicate mean (±95% CI) of the function that best described the response. Note that even though irradiance coding is a unique property of “sustained” neurons (Figure 6), a clear linear relationship between irradiance and firing rate is apparent in the pooled responses of “sustained” and “transient” cells in Opn1mwR mice. Thus, the deficiency of this activity in Opn4 −/− mice (B and Figure 6) does not merely reflect our inability to separate “sustained” and “transient” cell types in this genotype. (0.65 MB TIF) [file pbio.1000558.s008.tif]

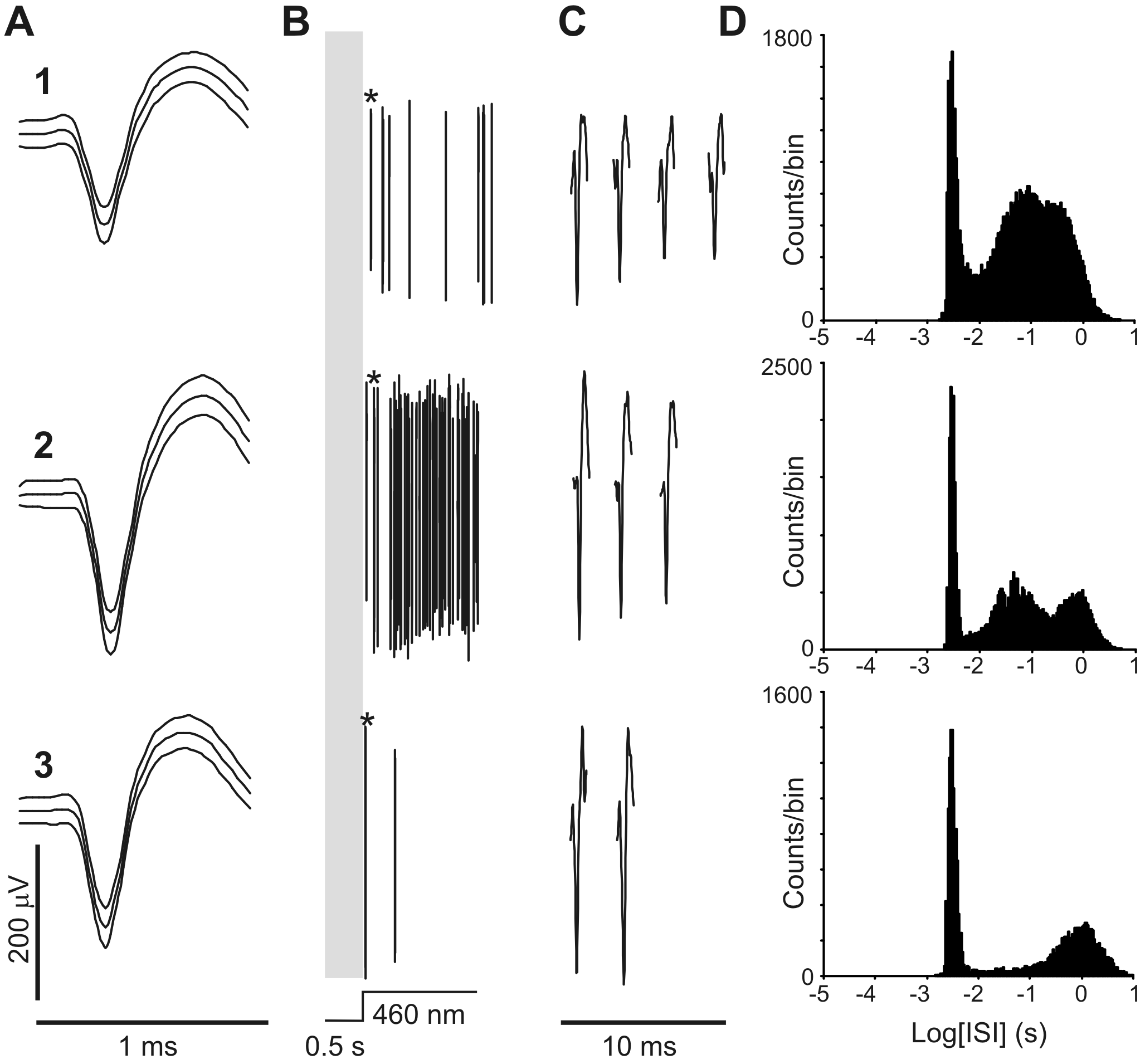

Supplement: Figure S9 — Single unit spike discrimination. (A) Mean (± SD) waveform of spikes assigned to three single units (corresponding to units 1–3 in Figure 4). (B) Spike patterns of single units from 0.5 s before to 1.5 s after the start of a 460 nm light pulse (8.3×10^14^ photons/cm2/s). (C) Expanded view of spike traces in (B) at the points marked *. (D) Log interspike interval (ISI) histograms for units 1–3. Histograms show a sharp peak at ISIs between 3 and 5 ms corresponding to spikes fired in bursts and broader peaks at longer ISIs corresponding to epochs of tonic firing. (0.26 MB TIF) [file pbio.1000558.s009.tif]
